# Nutrient availability influences *E. coli* biofilm properties and the structure of purified curli amyloid fibers

**DOI:** 10.1038/s41522-024-00619-0

**Published:** 2024-12-04

**Authors:** Macarena Siri, Mónica Vázquez-Dávila, Carolina Sotelo Guzman, Cécile M. Bidan

**Affiliations:** 1 Department of Biomaterials, Max Planck Institute of Colloids and Interfaces, Potsdam, Germany; 2 Max Planck Queensland Centre, Potsdam, Germany

**Keywords:** Biofilms, Biological techniques

## Abstract

Bacterial biofilms are highly adaptable and resilient to challenges. Nutrient availability can induce changes in biofilm growth, architecture and mechanical properties. Their extracellular matrix plays an important role in achieving biofilm stability under different environmental conditions. Curli amyloid fibers are critical for the architecture and stiffness of *E. coli* biofilms, but how this major matrix component adapts to different environmental cues remains unclear. We investigated, for the first time, the effect of nutrient availability both on biofilm material properties and on the structure and properties of curli amyloid fibers extracted from similar biofilms. Our results show that biofilms grown on low nutrient substrates are stiffer, contain more curli fibers, and these fibers present higher β-sheet content and chemical stability. Our multiscale study sheds new light on the relationship between bacterial matrix molecular structure and biofilm macroscopic properties. This knowledge will benefit the development of both anti-biofilm strategies and biofilm-based materials.

## Introduction

Bacteria co-exist in a community where they produce an extracellular matrix. Such living material is called a biofilm. Biofilm matrix typically contains DNA, exo-polysaccharides and proteins^1^. Such ecosystem is a form of organization that, among other functions, protects the bacteria from environmental challenges. Biofilm properties thus depend on environmental physical cues such as temperature, pH and water availability^2^.

The availability of nutrients is also proposed to play a major role in biofilm growth, morphology, mechanical properties and matrix composition^2–4^. For instance, nutrient limitations in the substrate might lead to spatial gradients of activities and molecules influencing the functioning of biofilms^5^. Variation of nutrient availability and their effect on different biofilm aspects have been studied for different bacteria such as *Bacillus sp*^4,6^., *P. fluorescence*^7,8^ and *V. fischeri*^9^. The development and composition of biofilms can be influenced by nutrient availability through (i) the variation of a specific nutrient^9^, (ii) the effect of metabolites in bacterial growth rate and mechanical properties^10^, (iii) alteration of the environment by metabolites and (iv) the addition of a substrate to promote the production of specific biopolymers by bacteria within the biofilm^9–11^. Nevertheless, how these variations affect the molecular properties of the bacterial matrix and impact biofilm properties remains to be understood.

In amyloid-containing biofilms, like in *E. coli* and *P. aeruginosa*, amyloid fibers are known to contribute to the backbone of the biofilm as they provide structural integrity^12–14^. In most cases, their molecular structure is influenced by the biofilm growth conditions^7,15,16^. There is also evidence of a correlation between the biofilm macroscopic properties (physico-chemical and mechanical) and the molecular composition and structure of the material (interactions and entanglement between the matrix polymers and charged smaller molecules)^2,7,16^.

Typically, amyloid fibers have a characteristic and stable quaternary conformation: cross-β structure with the β-strands arranged perpendicular to the fiber axis^17,18^. Their structure and fibrillation mechanism have been studied throughout the years under in vitro conditions^6,16,17,19^. Nonetheless, different studies describe not only an interaction between amyloids and components found in the biofilm matrix^15^, but also changes in the macroscopic features of the biofilm related to these protein assemblies^3,7,12,16^. Such evidence allows new questions regarding how the natural environment in which amyloids are fibrillated contribute to determine their final structure.

Nutrient diffusion and distribution through the biofilm result in cell differentiation patterns and subsequent stratification^20^. The molecular machinery responsible for amyloid fiber production in the biofilms made by *E. coli* and *S. typhimurium* is affected by stressful growth conditions^21^. Although much has been studied on the influence of environmental stress on biofilms, understanding the interplay between the structure/function of microbial matrix and the overall materials properties of the biofilm is still needed. Such knowledge could benefit both to biofilm prevention and to biofilm engineering for their use as bio-sourced materials.

This work focuses on the influence of nutrient availability on the structure of biofilm amyloid fibers and biofilm mechanics. We used the *E. coli* strain K12 W3110 that only produces curli fibers as the main component of its matrix, and cultured biofilms on agar substrates with different nutrient concentrations (i.e., yeast extract and tryptone). After 5 days, we quantified the size, mass and water content of the biofilms and estimated their mechanical properties by microindentation. Curli fibers were then purified from the biofilms, and fluorescence and ATR-FTIR spectroscopy were used to study the differences in their structure and functional properties like hydrophobicity and chemical stability. Overall, we provide knowledge to deepen the understanding of the relation between molecular features of the matrix components (e.g., fiber conformation) and macroscopic properties of the biofilm (e.g., stiffness).

## Results

Biofilm from *E. coli* K12 W3110 bacteria were grown on salt-free Lysogenic Broth (LB) agar substrates with different nutrient contents (0.75, 1.5, 3.0, 6.0 and 12.0% w/v) (Supplementary Fig. 1, Table 1). We analyzed the macroscopic features (i.e., size, mass, bacterial growth rate, total protein expression and biofilm stiffness) of the biofilms obtained in the different growth conditions. Curli fibers were then purified from the different biofilms and their structural and chemical characteristics were determined using spectroscopy techniques.

**Table 1:** Nominal nutrient contents respectively for the salt-free agar substrates in this work

Nominal agar concentration (w/v%)	1.8		1.8		1.8		1.8		1.8	
Nutrient concentration (w/v%)	0.75		1.5		3		6		12	
Nutrient (g)/100 mL										
*Tryptone: Yeast*	0.5	0.25	1	0.5	2	1	4	2	8	4
Effective water content (w/w%)	95.03		93.61		90.84		85.53		75.75	

Of note, the agar composition containing 1.5% nutrients is referred to as the standard biofilm growth condition because it has the same composition as the LB agar plates used to grow single bacterial colonies, without NaCl to promote biofilm formation^22^.

### Nutrient availability influences biofilm size, mass, matrix architecture and rigidity

**Fig. 1:**
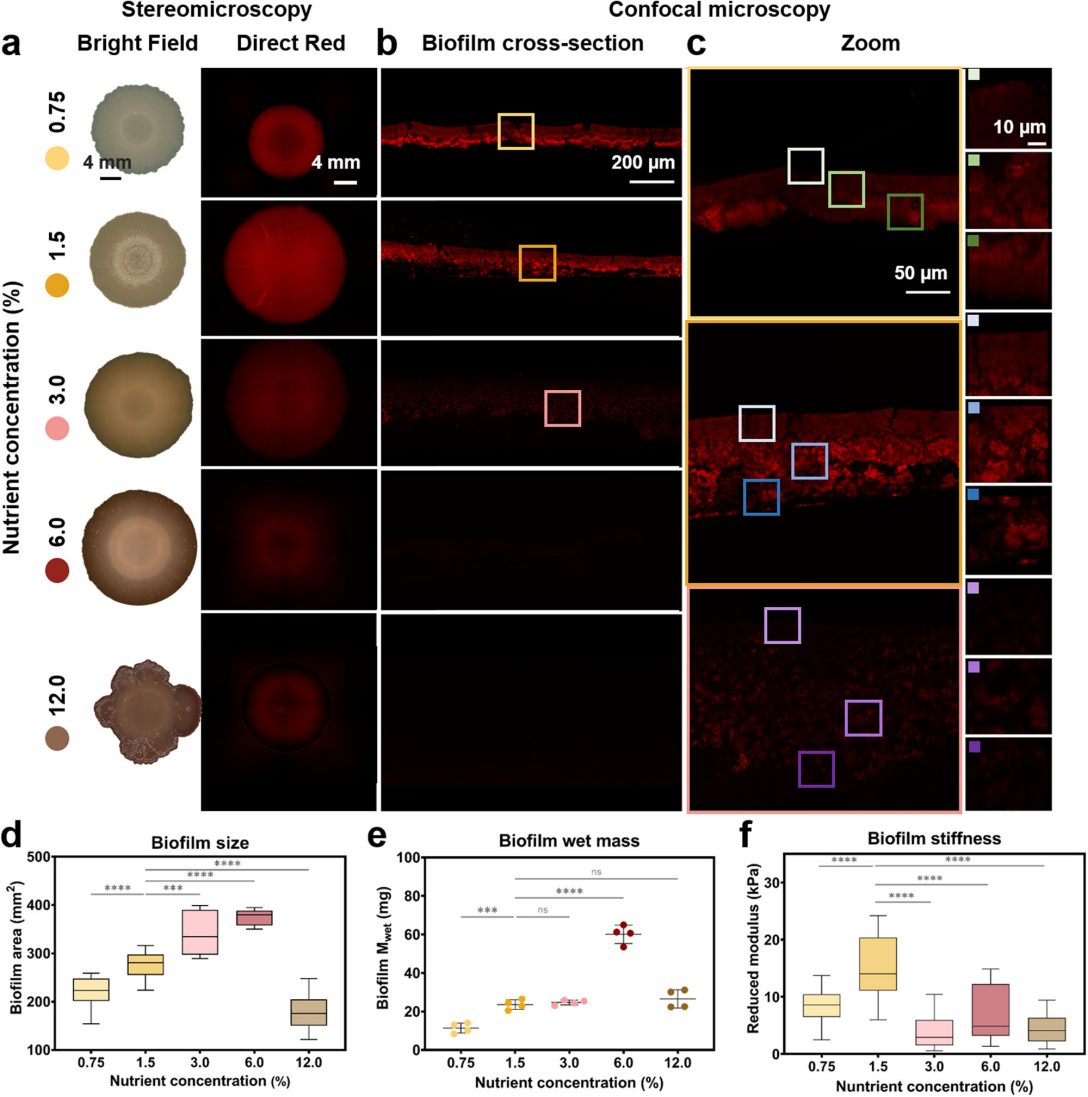
Macroscopic properties of *E. coli* W3110 biofilms in agar substrates with various nutrient concentrations. **a** Representative phenotype of 5-day old biofilms grown on substrates with different nutrient concentration bright field images and fluorescence images of the biofilms stained with Direct Red^23^. Scale bar = 4 mm. **b** Cross-section of the biofilms stained with Direct Red 23 and imaged by confocal microscopy. **c** Zoom of cross-sections of biofilm grown on agar containing 0.75–3.00% w/v nutrients. The respective area of the zoom of the cross-section are indicated by squares of each area. **d** Biofilm size based on their area. The statistical analysis was done with Mann–Whitney *U* test (*p* < 0.0001, **** | *p* < 0.001, *** | *p* < 0.01, ** | *p* < 0.05, * | ns = non-significant), where the 1.5% w/v nutrient concentration condition was used as reference. Data acquired from 15 independent biofilms per condition. **e** Biofilm wet mass. Values are calculated for a single biofilm from four independent experiments. The statistical analysis was done with One-way ANOVA (*p* < 0.0001, **** | *p* < 0.001, *** | *p* < 0.01, ** | *p* < 0.05, * | ns = non-significant), where the 1.5% w/v nutrient concentration condition was used as reference for the post-test multicomparisons. **f** Averaged reduced Young’s modulus obtained from microindentation experiments performed on biofilm surfaces. 10 − 23 individual measurements were done per condition. The statistical analysis was done with Mann–Whitney *U* test (*p* < 0.00 = 1, **** | *p* < 0.001, *** | *p* < 0.01, ** | *p* < 0.05, * | ns = non-significant), where the 1.5% w/v nutrient concentration condition was used as reference for the post-test multicomparisons.

Variations in the nutrient availability of the agar substrate yielded biofilms different in size, morphology and matrix arrangement (Fig. 1a–c and Supplementary Figs. 2–4). Staining curli with the fluorescent dye Direct Red 23 (dye also known as Pontamine Fast Scarlett 4b)^23^ not only showed differences of matrix distribution (Fig. 1a–c) in the cross-sections, but also a clear decrease of dye intensity in biofilms grown in nutrient-rich conditions, suggesting differences in overall curli content in their matrix and/or changes in the interaction between dye and fibers (Fig. 1a–c and Supplementary Figs. 3, 4).

The area of biofilms increased from 220 ± 32 mm^2^ for substrates containing 0.75% w/v nutrients, to 374 ± 16 mm^2^ for substrates containing 3.0% w/v nutrients. The biofilm size decreased for biofilms grown on salt-free LB agar substrates containing higher nutrient content, reaching a size of 178 ± 35 mm^2^ on substrates containing 12.0% w/v nutrients (Fig. 1b, c and Supplementary Fig. 2). This last condition also yielded biofilms different in shape (Fig. 1a (right panel) and Supplementary Fig. 2).

Along with their size, changes in the biofilm wet mass (M_wet_) were quantified by weighing (Fig. 1d and Supplementary Table 1). Biofilms grown on salt-free LB agar substrates containing 6.0% w/v nutrients showed the highest wet mass, while biofilms grown at low nutrient availability (0.75% w/v) presented the lowest wet mass (Fig. 1e). The rest of the conditions resulted in biofilms with similar wet mass. A similar trend was observed for the estimated dry mass in the biofilms (M_dry_) (Supplementary Fig. 5 and Supplementary Table 1).

We carried out microindentation experiments to study whether these changes in size and wet mass had an effect on the stiffness of the biofilms (Fig. 1f and Supplementary Fig. 6). The biofilm stiffness was estimated by indenting up to 10 μm in the center of the biofilm (10% of its thickness approximately) to avoid influence of the substrate during the measurement (Supplementary Fig. 6, inset). Biofilms grown on substrates containing 1.5% w/v nutrients presented the highest stiffness value, 15 ± 5 kPa (Fig. 1f). Biofilms grown on substrates containing 3.0 and 12.0% w/v nutrients showed the lowest values. Results suggest that the stiffness from the biofilm is independent of their size or their wet mass.

Fluorescence imaging performed on biofilm cross-sections (Fig. 1b) revealed differences in the matrix arrangement, highlighting biofilm heterogeneity. While the obtained stratified organization aligns with the literature^20^, the layer of bacteria deprived of matrix and in direct contact with the agar is not clearly observed in the conditions with low nutrient availability (Fig. 3b). Such a stratified structure also suggests that the stiffness obtained by microindentation mainly corresponds to the stiffness of biofilm upper layer.

Biofilms grown on agar substrates with lower nutrient content exhibit a matrix arranged into small patches in two layers of different densities within the fluorescent layer of the biofilm (0.75 and 1.5% w/v nutrients). As the nutrient availability increases, the biofilm matrix loses the patch arrangement in favor of a more homogeneous distribution. The other biofilm properties presented in this work are averaged over the whole biofilm and such heterogeneity calls for caution when using these data to infer bacteria behavior.

### Nutrient availability influences biofilm composition and ability to uptake water

**Fig. 2:**
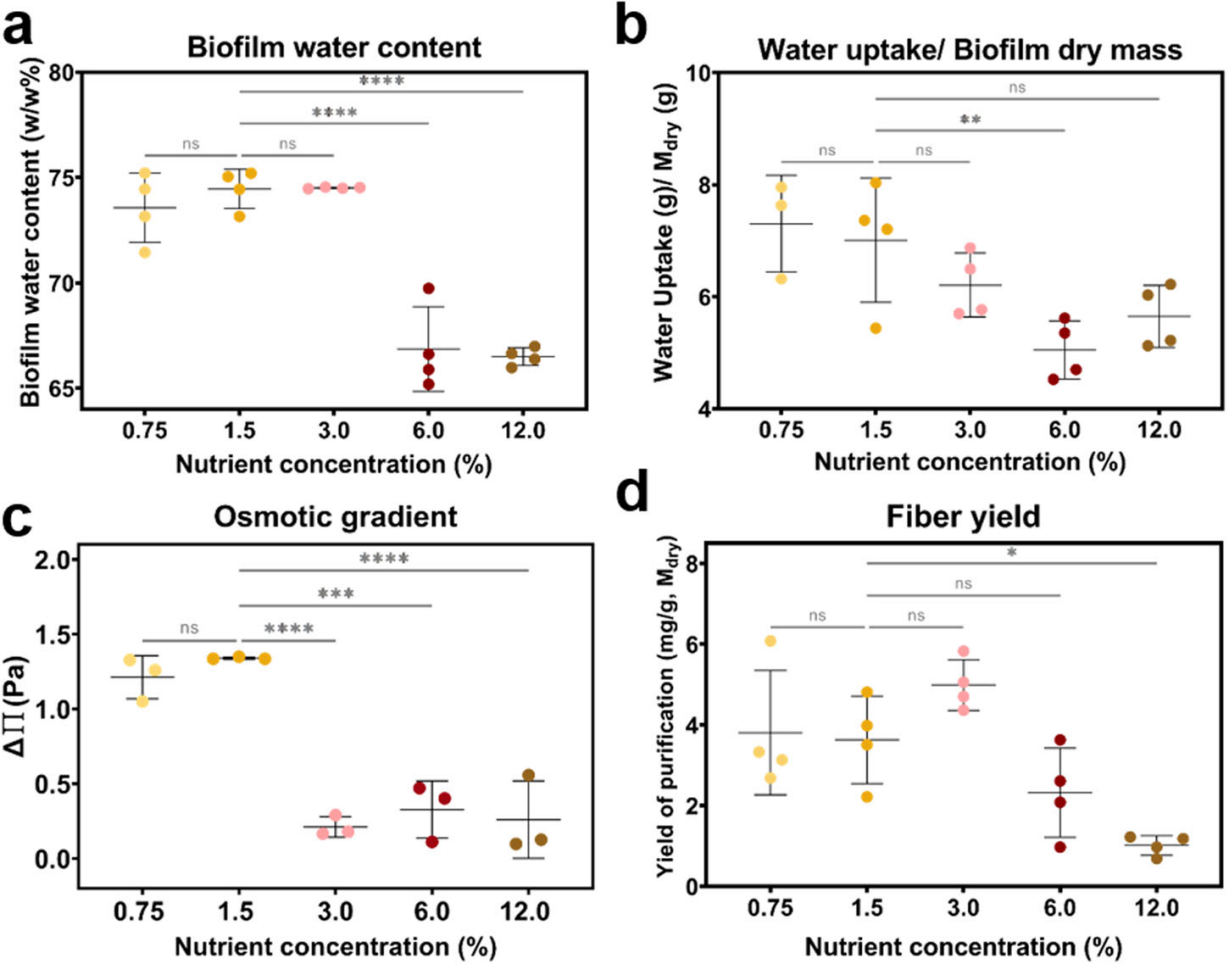
General characteristics of the composition of *E. coli* W3110 biofilms grown on substrates with different nutrient contents. **a** Biofilm water content. **b** Biofilm water uptake per gram of dry mass. **c** Osmotic gradient between the biofilm and the agar substrate. **d** Purification yield of the curli fibers extraction process in milligram of CsgA per gram of dry mass. All data presented here come from *N* = 4 independent biofilm cultures for each condition tested. The statistical analysis was done with One-way ANOVA (*p* < 0.0001, **** | *p* < 0.001, *** | *p* < 0.01, ** | *p* < 0.05, * | ns = non-significant), where the 1.5% w/v nutrient concentration condition was used as reference for the post-test multicomparisons.

Biofilms were further characterized to estimate their composition, including their water content and their water uptake ability (Fig. 2 and Supplementary Table 1). The water content was assessed by weighing the biofilms before and after dehydration. Biofilms grown at low nutrient availability (0.75–3.0% w/v) presented the highest water content c.a. 74% w/w, while biofilms grown at high nutrient availability presented the lowest water content, approximately 66% w/w (Fig. 2a). The water uptake ability of the biofilms was tested upon overnight rehydration (Supplementary Fig. 7). Except for biofilms grown on salt-free LB agar containing 3.0 and 6.0% w/v nutrients, all biofilms presented a similar rehydration behavior. Biofilms grown at low nutrient availability (and at 12.0% w/v nutrient concentration) absorbed 55–70% more of water than their original weight. Biofilms grown on salt-free LB agar containing 3.0 and 6.0% w/v nutrients absorbed 30% of water more than their original weight. Analysis of the water uptake per gram of dry biofilm after rehydration showed that, the lower the nutrient availability, the higher the water uptake (Fig. 2b).

To deepen our understanding on how the biofilm matrix changes with biofilm growth conditions, we focused on the biofilm composition and first estimated the total amount of proteins using a Bradford assay (Supplementary Fig. 8). Alongside, we studied the growth of bacteria in liquid media of different nutrient compositions to clarify whether the differences observed were influenced by their metabolism (Supplementary Figs. 8–10). The results show that although the bacteria had a higher metabolism in higher nutrient media, the protein production did not follow this trend. While these results are useful to apprehend bacteria metabolism, one should remember that from the physiological point of view, liquid cultures are very different from the biofilm lifestyle.

The growth and spreading of biofilms need bacteria to consume water and nutrients from the agar substrate and previous studies proposed osmotic pressure gradients (∆∏) to support such transport^24,25^. We thus calculated these gradients from water activity measurements in the agar substrates and the corresponding biofilms (Fig. 2c). Biofilms grown on agar substrates with low nutrient content (0.75 and 1.5% w/v) present the highest osmotic gradients around 1.3 MPa and therefore the highest water influx. In contrast, biofilms grown on agar substrates with higher nutrient content (3.0–12.0% w/v) show small osmotic gradients around 0.3 MPa.

Among the proteins present in the biofilm, curli amyloid fibers are the main components in the biofilm matrix. We purified them and estimated a yield by quantifying the monomeric unit (CsgA) and normalizing per gram of dry biofilm (Fig. 2d). Interestingly, biofilms grown on salt-free LB agar substrates containing 12.0% w/v yield two times less purified curli fibers compared to the other conditions. No significant differences were observed for the other yields in fiber production. We observe that the curli fibers yield does not follow the trend of the biofilm total protein concentration, nor the trend of the bacterial metabolic activity. However, there is a tendency for increased biofilm water uptake at higher fiber yields.

### Nutrient availability influences the packing and structure of curli amyloid fibers in the biofilms

**Fig. 3:**
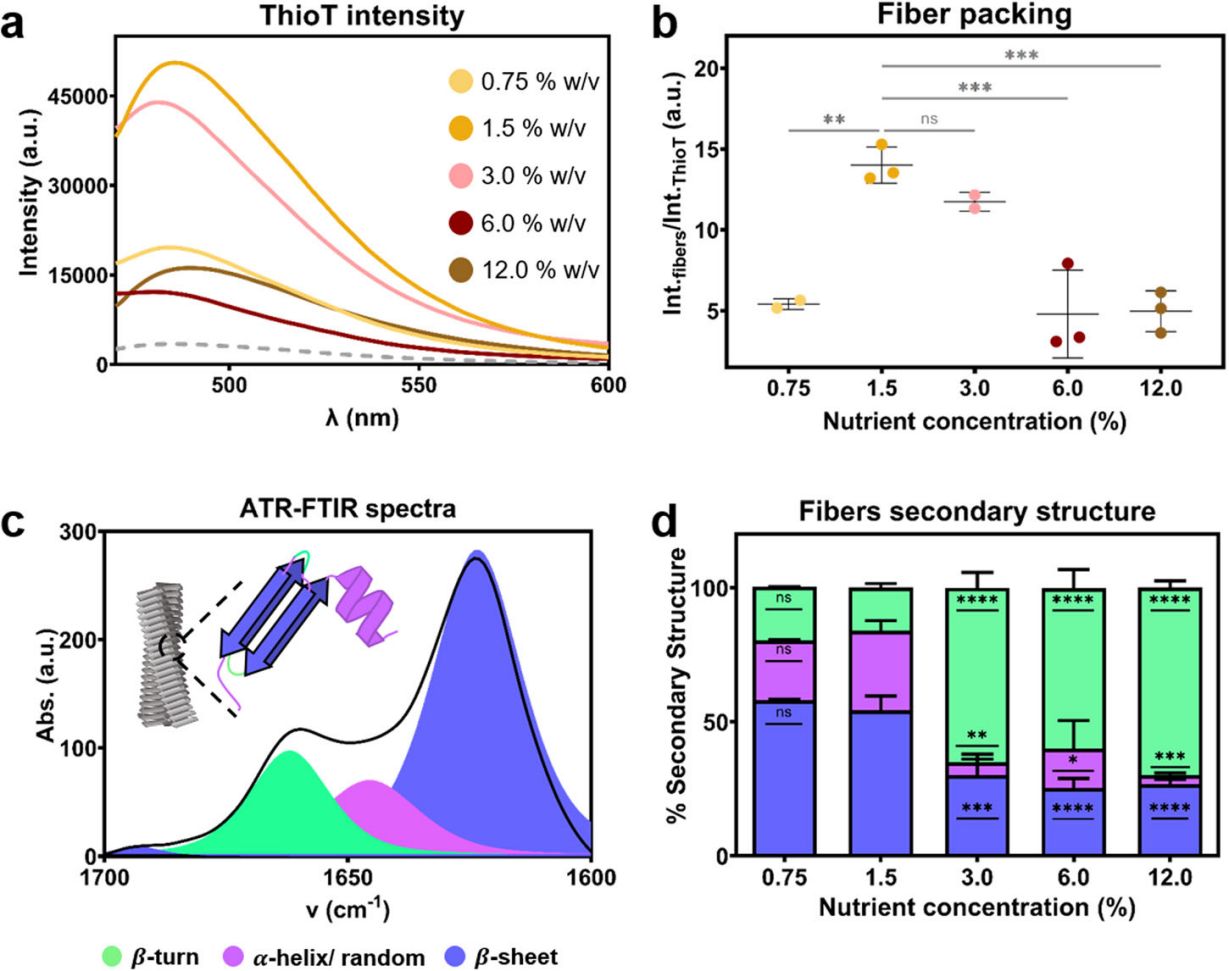
Characterization of the structure of purified curli fibers. **a** ThioT fluorescence emission spectra of the fibers at equivalent mass concentrations. **b** Values describing the increase in intensity of ThioT when bound to the purified fibers. Quantification of the increase was estimated by division of the area under the curve of each spectra of the probe with each fiber by the area under the curve of the emission spectra of the probe alone. The statistical analysis was done with One-way ANOVA (*p* < 0.001, *** | *p* < 0.01, ** | *p* < 0.05, * | ns = non-significant), where the 1.5% w/v nutrient concentration condition was used as reference for the post-test multicomparisons. **c** Representative Amide I’ region of an ATR-FTIR spectrum for curli fibers. Each peak assigned to the different secondary component is highlighted in their respective color. **d** Distribution of the three types of secondary structure in the curli fibers obtained in the different growth conditions. The data was obtained from the Amide I’ region of each spectra. The statistical analysis was done with One-way ANOVA (*p* < 0.0001, **** | *p* < 0.001, *** | *p* < 0.01, ** | *p* < 0.05, * | ns = non-significant), where the 1.5% w/v nutrient concentration condition was used as reference for the post-test multicomparisons.

The structure of curli fibers after the purification process was examined by transmission electron microscopy (TEM), by Thioflavin T fluorescence emission (ThioT) and by Fourier transform infrared (ATR-FTIR) spectroscopy (Fig. 3a–c). TEM and SDS-PAGE confirmed the presence of curli fibers in the purified product (Supplementary Figs. 11, 12). Following basic amyloid fiber characterization, we stained the fibers with ThioT. This dye undergoes a fluorescence enhancement when immobilized in the β-sheets of the amyloid fibers due to binding^26^. Despite every sample having ThioT fluorescence emission, there were differences in the intensity values (Fig. 3a). When normalizing the intensity value against the free ThioT signal, fibers produced on substrates containing 1.5 and 3.0% w/v of nutrient showed an increase of c.a. 15 times of the ThioT intensity (Fig. 3b). Fibers produced in a context of higher or lower nutrient availability showed only a five-fold increase in the ThioT intensity. The differences observed here suggest differences in the packing or in the β-sheet content of the fibers^16^. Similar results were observed as we identified the fibers with congo red staining (Supplementary Fig. 13).

ATR-FTIR spectroscopy was used to further study the fiber structure (Fig. 3c, d). We focused on the amide I′ band (from 1700 to 1600 cm^−1^), i.e., the fingerprint region for protein analysis (Fig. 3c)^27–29^. All samples showed spectra expected for curli amyloid fibers (Fig. 3c and Supplementary Fig. 14): a band around 1620 cm^−1^ assigned to $\beta$-sheet structures, and second band around 1660 cm^−1^ usually assigned to $\beta$-turns structures^16,17^. The intensity and width of the band at 1660 cm^−1^ increased with the nutrient availability, indicating differences in the secondary structure composition of the fibers (Supplementary Fig. 14). We used a band fitting method to decompose the spectra into the secondary structure components (Table 1)^16,27^. Purified curli fibers obtained from biofilm grown at low nutrient availability (0.75 and 1.5% w/v) had the highest content of β-sheet and the lowest content of β-turn structures (Fig. 3d and Supplementary Table 2). In contrast, purified curli fibers obtained from biofilm grown at higher nutrient availability (3.0–12.0% w/v) had the lowest content of β-sheet and the highest content of β-turn structures (Fig. 3d and Supplementary Table 2). Differences in the β-sheet structure of the fibers was also observed by CD spectroscopy (Supplementary Fig. 15). Once bound to the fibers, ThioT informs on the exposed β-sheets and/or the spacing between them^30^. The differences in β-sheet amount revealed by ATR-FTIR and CD spectroscopy do not follow the trend reported by the ThioT experiment. This suggests that here, ThioT mainly reports differences in the spacing between β-sheets (fiber packing)^16^.

### Nutrient availability influences curli physico-chemical features in the biofilms

**Fig. 4:**
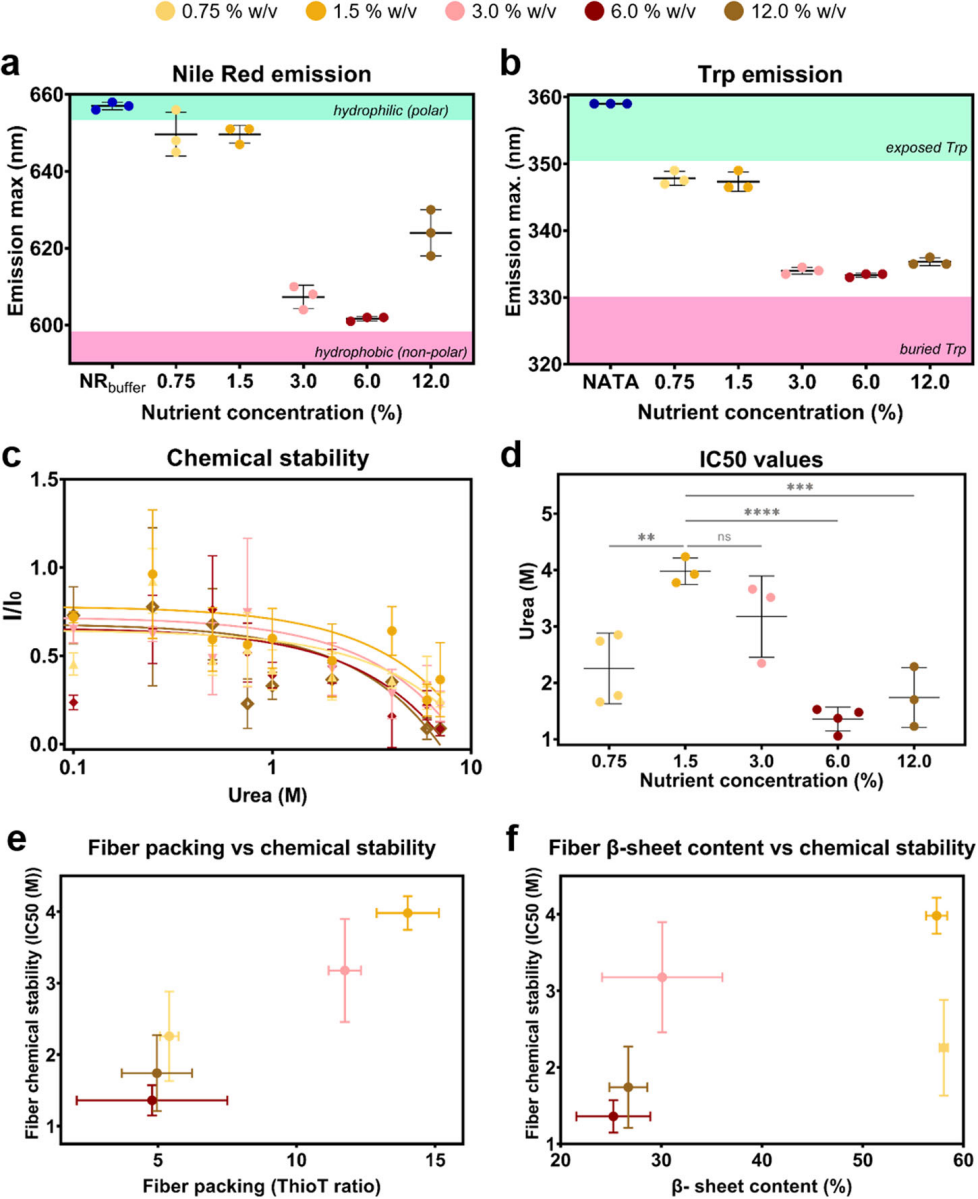
Structure-function features of the purified curli fibers. **a** Position of the emission peak of the fluorescence spectra of fibers stained with Nile Red (NR). The spectra of NR in buffer is represented as a reference for the emission of NR in a hydrophilic environment. The shadowed areas in the main plot indicate these exposure extremes. *N* = 3–4 independent biofilm cultures for each condition tested. **b** Position of the emission peak of the intrinsic fluorescence of the fibers through Trp emission. The spectrum of soluble Trp (NATA) in buffer is represented as reference for the maximum exposure possible of the Trp to the surface (λ_exc_ = 280 nm). The shadowed areas in the main plot indicate these exposure extremes. *N* = 3–4 independent biofilm cultures for each condition tested. (For the spectra of (**a**) and (**b**), please refer to Supplementary Fig. 10). **c** Chemical stability of the purified fibers upon denaturation with increasing urea concentrations (0.1–8 M). The presence of the fibers was observed by ThioT fluorescence emission intensity. I_0_ corresponds to the ThioT emission when bound to fibers without urea in the solution. **d** IC50 values for each curve in panel (**c**) IC50 corresponds to the urea concentration at which the ThioT intensity is 50% of the initial one. **e** Relationship between fiber packing (ThioT ratio) and their chemical stability. **f** Relationship between fiber β-sheet content and their chemical stability. *N* = 6 independent fiber samples. The statistical analysis was done with One-way ANOVA (*p* < 0.0001, **** | *p* < 0.001, *** | *p* < 0.01, ** | *p* < 0.05, * | ns = non-significant), where the 1.5% w/v nutrient concentration condition was used as reference for the post-test multicomparisons.

Since molecular properties and functions are related to structure, we explored if the differences observed on the fiber conformation are reflected in their physico-chemical features. The global hydrophobic character of the purified curli fibers was studied by binding to Nile Red (NR) (Fig. 4a and Supplementary Fig. 16a). NR is a solvatochromic dye reporting the hydrophobic character of the molecule it binds to. We used the position of the peak of the emission spectrum of NR in buffer as references to assess the hydrophobic character of the amyloid fibers obtained in the different conditions (Fig. 4a). All purified fibers present a strong hydrophobicity. However, as the nutrient availability in the substrate increased, the hydrophobic character of the fibers also increased (Supplementary Fig. 16a, c).

Curli fibers are made of CsgA monomers, each of them containing a single tryptophan (Trp) (Supplementary Fig. 17). Trp is an amino acid behaving as a solvatochromic fluorophore (Supplementary Fig. 16b, d)^16^, which position of maximum fluorescence emission gives information on the hydrophobicity of its environment (Fig. 4b)^31^. Curli fibers grown at a low nutrient concentration (0.75% and 1.5% w/v) presented a Trp population in a less hydrophobic environment than the other fibers.

Finally, we studied the implication of the different structures of purified curli fibers by testing their chemical stability (Fig. 4c, d). Fiber denaturation into CsgA monomers was monitored by the fluorescence intensity emitted by ThioT^16^. Curli fibers obtained from biofilms grown on salt-free LB agar containing 1.5% w/v nutrients had the highest stability, while fibers grown at lower nutrient availability (0.75% w/v) or higher than 3.0% w/v presented low chemical stability (Fig. 4c). Fibers with a high packing degree and a high β-sheet content, presented high chemical stability (Fig. 4e, f)^16,32^. The higher the packing in the fiber, the higher the chemical stability. While biofilm stiffness may be influenced by different fiber properties, no further particular trend emerged (Supplementary Fig. 18).

## Discussion

**Fig. 5:**
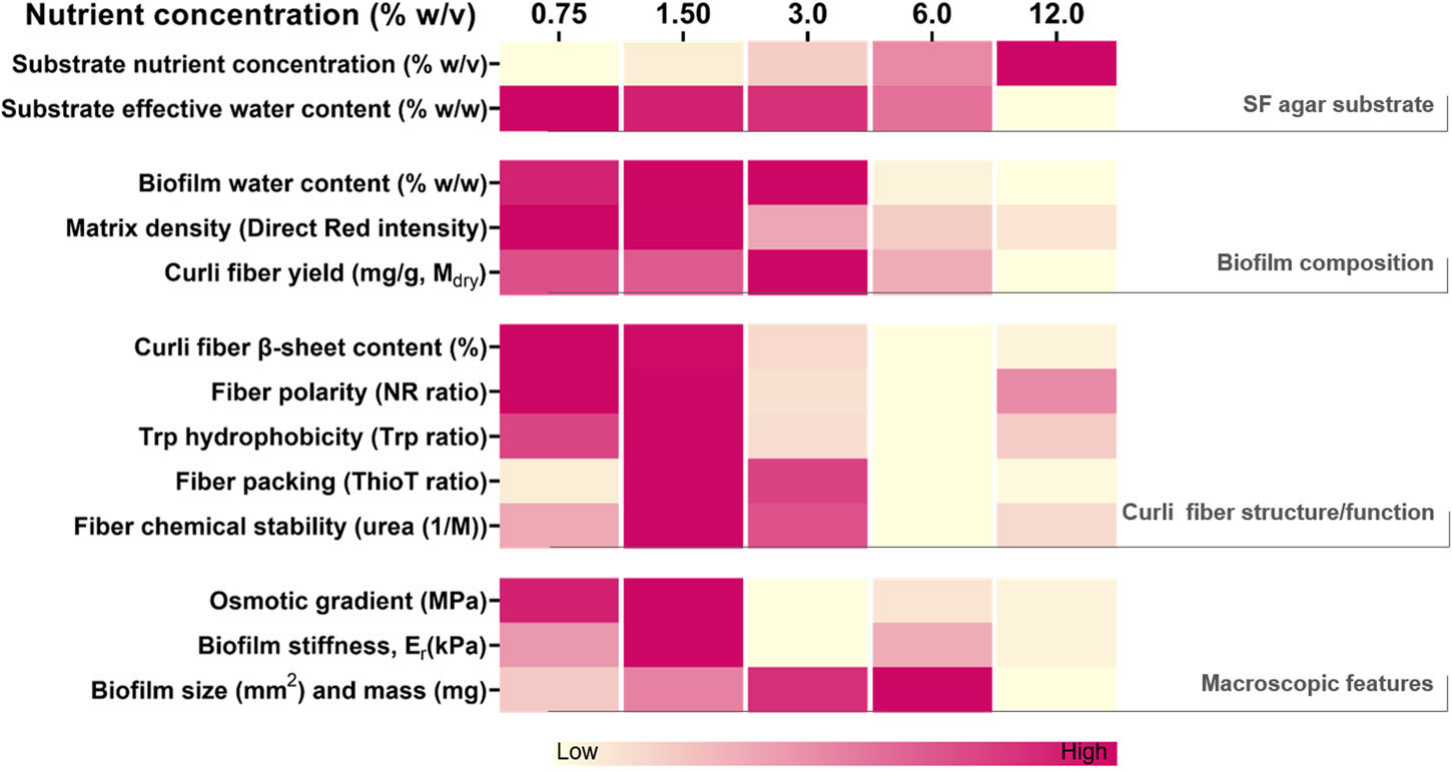
Summary of the relationship between nutrient variation in the salt-free LB agar and the macro- and microscopic features of the biofilms grown under each condition. The Supplementary Figure serves as a visual global analysis of the data acquired and guide to the following discussion integrating the results obtained in this work. We indicate in color shade the position of the parameter value in the column on the left of one of the conditions compared to the other conditions studied (yellow for the lowest value, to fuscia for the highest value).

Our previous work investigating the influence of water availability on the structure of curli amyloid fibers and its implications in the macroscopic biofilm characteristics, revealed that lower water availability in the substrate led to stiffer *E. coli* biofilms containing curli fibers with higher ß-sheet content^16^. While the exact mechanisms are still not clear, it was proposed that water availability indirectly influences bacteria metabolism through its important role in nutrient supply. In the present work, we fixed the agar content and the volume of water in all the substrates, and only varied the nutrient concentration. We then studied how nutrient availability influences *E. coli* biofilms from the macroscopic to the molecular scale (Fig. 5). The results show that biofilm size, mass and rigidity change as the amount of surrounding nutrients varies (Fig. 1). Such effects of yeast extract and tryptone variations on macroscopic biofilm properties (e.g., morphology, thickness and density) have been reported for other bacteria strains^4,9,33^. In the case of *E. coli* K12 W3110, we observed that the relation is not monotonic and rather shows a maximum of these properties at intermediate nutrient availability, namely 6.0% w/v for the size and mass and 1.5% w/v for the stiffness (Fig. 1). The existence of such optima may result from simultaneous and opposite effects of substrate properties that cannot be decoupled from the nutrient content. For example, both the presence of nutrients and water are expected to promote biofilm growth respectively *via* the stimulation of bacterial activity, the lubrication of the surface and biofilm swelling^24,25^, yet salt-free LB agar containing higher amounts of nutrients also happen to contain less free water (Supplementary Fig. 1a–c), which can constitute a stress impairing bacterial growth (e.g., at 12.0% w/v). Nutrient availability has also proven to be as important at the initial steps of the biofilm formation e.g., in the layout of the bacteria^34^, as at the later stages in which water channels form for nutrient distribution^11^, and gradients are established^35^. The implication of nutrients at different hierarchical levels of the biofilm architecture can thus explain the effect of their concentration on a broad range of biofilm properties (Fig. 5)^36^.

As we decomposed the biofilm mass obtained into the contributions of water, bacteria and curli fibers, we observed that biofilm water content and curli fiber yield both tend to be higher when nutrients are less abundant (Fig. 2a, d). Although not the sole factor, amyloid fibers are known to contribute to keeping a hydrated microenvironment within biofilms^37^. This can be explained by the potential of amyloid fibers to take up and bind water (Fig. 2b, c)^38^. Our work suggests that this potential is enhanced when curli fibers are extracted from biofilms grown in environments with limited nutrients, as indicated by their higher polarity (Fig. 4a).

More generally, the water uptake of biofilms is regulated by the composition and characteristics of their extracellular matrix^39^. Indeed, biofilm matrix influences the osmotic pressure driving water flows leading to nutrient transport and subsequent bacterial growth, but also to biofilm expansion *via* matrix swelling, as showed for *B. subtilis* and *V. cholerae*^25,39,40^. Our results show that tuning biofilm matrix properties can also be used by *E. coli* bacteria to create osmotic gradients (∆∏) able to promote the transport of nutrient-rich water from the environment to the biofilm (Fig. 2c). This mechanism in turn supports both biofilm growth through bacteria proliferation and biofilm expansion through swelling. Osmotic gradients most probably also influence matrix architecture within the biofilm, as suggested by the presence of matrix in direct contact with the agar substrate in growth conditions where ∆∏ values are higher (Supplementary Fig. 3b) i.e., in nutrient-poor conditions, and the loss the curli patch arrangement in growth conditions where ∆∏ values are lower (Figs. 1a and 2c), i.e., in nutrient-rich conditions. We can speculate that bacteria growing in such abundant conditions favor proliferation (Supplementary Fig. 10) at the expense of matrix production (Fig. 2d), as they have less pressure to create the required influx to supply them with nutrient. This behavior could partly explain the bulky morphology of biofilms obtained from nutrient-rich conditions (12.0% wt), i.e., thick biofilms with limited spreading (Fig. 1a, b). In contrast, the first generations of bacteria grown in a nutrient-poor environment may rapidly onset matrix production in order to create the osmotic gradient necessary to get water flowing towards the biofilm, and thus insure nutrient supply. As a result, this experimental study varying nutrient concentrations supports the proposed explanations on how water availability in agar substrates could influence *E. coli* biofilm matrix at different length scales^16,41^.

The trend of higher curli fiber yield when nutrients are less abundant is also consistent with bacteria having less metabolic and proliferation activities in such conditions (Supplementary Fig. 10). The nutrient concentration and more generally the composition of the substrate influence the composition of the biofilm matrix and subsequently biofilm properties^4,9,11^. In *B. subtilis* biofilms for example, the composition of the growth medium was shown to actively contribute to the production, the distribution and the characteristics of the amyloid fibers found in the extracellular matrix^4^. *V. fischeri* biofilms also acquire different morphologies and resistance to mechanical disruption depending on the nutrients available to the bacteria^9^. Interestingly, bacteria grown on agar with the highest nutrient content (12.0% w/v) produced smaller biofilms containing less curli fibers (Fig. 2d). This observation is consistent with the general understanding of matrix secretion and subsequent biofilm formation as part of the multiple bacteria responses to starvation^37,42^. In such extreme nutrient-rich condition, we cannot exclude the onset of osmo adaptation mechanisms by *E. coli* to adjust to relatively high osmotic pressures compared to their ideal (300 mOsmol/kg, i.e., a_w_ = 0.9946). We measured the water activities in this substrate and in the corresponding biofilms to be 0.9885 and 0.9859, respectively, which is largely above the minimum value of 0.95 required for *E. coli* growth. A transient slowdown of growth upon seeding bacteria in such conditions is expected due to the onset of osmolarity regulation mechanisms, but growth arrest is not foreseen as long as nutrient supply is guaranteed. Here, the osmotic increase being relatively mild and due to a high concentration of nutrients, we can assume that (i) osmo adaptation will be quick^43^, (ii) a shortage of energy supply may arise after a few days of growth, and (iii) limited energy will be spent on matrix production. This would explain the thick biofilms with low signal of direct red (Fig. 1a) as well as the low curli purification yield obtained in this condition (Fig. 2d).

Biofilms can be considered as hydrogels and their mechanical properties are expected to be determined by both their network of biopolymers and their water content^5^. Here we observe the counterintuitive result that biofilm stiffness is higher in conditions where biofilm water content is larger (Figs. 1d and 2a). This can be explained by a denser biopolymer network as indicated by the higher Direct Red signal and the larger yield of curli fibers obtained in these conditions (Figs. 1a and 2f). Beyond their amount, curli fibers are likely to contribute to the macroscopic mechanical properties of the biofilms through their molecular structure and supra-molecular arrangement when forming the matrix (Fig. 1a and Supplementary Fig. 18). Indeed, the arrangement of dense patches of matrix observed in biofilms grown in nutrient-poor conditions can explain the higher moduli measured by microindentation. Moreover, curli fibers themselves are known for the relatively high rigidity provided by the cross-β sheet arrangement adopted by the CsgA subunit^17,19,44^. Variations in the conformation of the amyloid proteins are thus expected to induce variations in the rigidity of the fibers, which in turn are expected to influence biofilm stiffness. Consistently, analyzing the structure of the curli fibers extracted from biofilms obtained in the various conditions revealed a relatively higher packing and β-sheet content, when obtained from the stiffest biofilms, i.e., at 1.5% w/v nutrient concentration (Figs. 2d, 3b and 3d, and Supplementary Figs. 14, 15). We interpret the ThioT ratio of each fiber as fiber packing due to a combined interpretation of the ATR-FTIR spectroscopy, CD spectroscopy and intrinsic fluorescence analysis (Fig. 3d, Supplementary Figs. 16 and 4b). Specifically, the curli fibers that showed low ThioT intensity presented a low β-sheet content with ATR-FTIR and CD spectroscopy as well as change in the Trp environment. Combined, these results suggest that ThioT signal could be describing the fiber packing^26^. These structural variations could also explain the different fluorescence emission spectra in the presence of Nile Red, and thus the different amyloid fiber polarity measured as a function of nutrient availability during biofilm growth (Fig. 4a and Supplementary Fig. 16a)^45^. The same reasoning can apply to the Trp amino acid present in the CsgA subunits, which intrinsic fluorescence indicates the hydrophobicity of its close environment (Fig. 4b, and Supplementary Fig. 16b)^31^. Of note, the changes observed in the low β-sheet content of the curli produced at higher nutrient concentrations (3.0–12.0% w/v) could also explain the low emission of the dye’s intensity when observing the biofilm matrix arrangement (Figs. 3d and 1a–c). The changes in the fiber structure could have as a result a lower interaction with the dye. Testing the integrity of the different samples in the presence of urea also revealed that the chemical stability of curli increases with fiber packing, including an additional influence of their β-sheet content (Fig. 4d–f). Considering the mode of action of urea, this result is consistent with the idea that the access of urea to the peptide is easier in loosely packed fibers, thus facilitating amyloid denaturation^16,46^. It is interesting to note that the fibers produced by biofilms grown on low nutrient agar substrates (0.75 and 1.5%w/v) show a variation in their packing but not in their secondary structure. This result suggests that in this case, it is the environment, rather than the bacteria, that influences fiber physico-chemical properties.

The multiple experiments presented in this work converge to the major finding that nutrient availability in the surroundings of the bacteria affects not only the production, but also the structure of the curli amyloid fibers they produce (Fig. 5). As this result may appear surprising in light of the existing literature on the bacterial amyloid curli, it is important to highlight that the structural investigations reported here were performed on amyloid curli fibrillated in vivo, i.e., in the native biofilm micro-environment^15^. The present physico-chemical context is thus significantly different, which may have a substantial impact on the resulting fiber conformation. For example, bacterial cell membranes, as well as the presence of lipopolysaccharides (LPS) may promote fiber aggregation^15^. The matrix molecular crowding, especially the presence of extracellular DNA, is also contributing to the fibrillation process of amyloid fibers^15^. Other environmental conditions, such as pH were also shown to influence this process (although in vitro)^17^. In a broader context, exopolysaccharides present in many biofilm matrices, such as phosphoethanolamine-cellulose in *E. coli*^47^, may not only have great influence on amyloid assembly, but they may also be influenced by nutrient availability during biofilm growth. Further investigations will be needed to understand this complex interplay between different microbial matrix fibers as a function of the biofilm environment. Such knowledge may become of high interest to compare and interpret biofilm characteristics obtained from different laboratories, i.e., in slightly different growth conditions.

Although as observed by the ∆∏ experiments we cannot fully decouple the role of nutrients and water in the biofilm development, we can notice some trends in behavior when the effect of both are studied separately. Previous works published by this group studied the effect of water availability in the substrate on the biofilm development^16,33^. Specifically, when comparing how the water availability affected the structure of curli amyloid fibers^16^ to the results in this work, we can observe that nutrient availability has a higher impact on the β-sheet content of the fibers. Higher water content in agar render fibers with slightly less β-sheet content, while here we can observe that the content of β-sheet in the curli amyloid fibers decreased abruptly when the nutrient concentration was doubled in the substrate (Fig. 3d). The abrupt changes in the structure of curli amyloid fibers due to nutrient availability can also be observed when studying their hydrophobicity character (Fig. 4a, b). Indeed, water seems to influence the biofilm spreading and size in *E. coli* biofilms to a higher extent than nutrient availability does (Fig. 1d)^16,33^. Moreover, the effect water has on the biofilm mechanics seems to be more clear than the effect nutrients have of the mechanical properties of *E. coli* W3110 biofilms (Fig. 1f).

Considering the protective function of biofilm matrix in challenging environments, one can question whether these variations in curli structure and their implications at the biofilm scale are the result of incidental physico-chemical interactions during the fibrillation process, or whether *E. coli* bacteria actively tune this process to provide amyloid curli fibers with specific properties (e.g., polarity, chemical stability, stiffness) in order to support the survival of the community (e.g., nutrient storage and transport, water retention, physico-chemical shield)^37^. An attempt to address this point with further experiments indicates that these two hypotheses are not necessarily exclusive (Supplementary Fig. 19). Indeed, after denaturing^48^ the curli fibers and repolymerizing the CsgA units in different media, the overall structural conformations of the original fibers obtained from biofilms grown in different conditions was retained (Supplementary Fig. 19g, h). Bacteria may thus produce CsgA monomers that are slightly different (probably in structure) depending on the matrix fiber structure and properties they need to cope with the given environment. Nevertheless, curli fibers reconstituted from CsgA monomers purified from biofilms grown in abundance of nutrient (LB 6% w/v) also showed structural variations depending on the re-fibrillation conditions (Supplementary Fig. 19g, h). Although the present approach allowed us to establish a proof of feasibility, another approach yielding more purified curli fibers is required to efficiently tackle the related questions about CsgA structure, adaptability, and curli biogenesis.

In general, the fundamental outcomes of this work can have great implications for the development of both anti-biofilm strategies and biofilm-based materials. Indeed, we found out that *E. coli* form overall weaker biofilms when exposed to environments that are either very poor or very rich in nutrients, mainly by altering the amount, packing and conformation of the amyloid curli fibers constituting their extracellular matrix (Fig. 5)^42^. This information could possibly motivate an overfeeding of bacteria coupled with antibiotic treatments to prevent the formation of strong biofilms and simultaneously target the vulnerable bacteria^49^. In contrast, the relatively more robust curli fibers harvested from biofilms formed by *E. coli* exposed to intermediate nutrient levels may represent building blocks with high potential for making amyloid-based materials^50^. The possibility of tuning properties of the bacterial amyloids *via* the conditions of their genesis may even expand the range of possibilities in the emerging field of engineered living materials^51^.

## Methods

### Agar substrate plate preparation

Salt-free agar plates (15 mm diameter) were prepared with 1.8% w/v of bacteriological grade agar−agar (Roth, 2266), supplemented with different concentration of tryptone (Roth, 8952) and yeast extract (Roth, 2363) (Table 1). Each agar plate was left to rest for 48 h before bacteria seeding to ensure the correct evaporation of possible water excess. After 5 days at 28 °C, the agar plates were ready for characterization.

#### Thermal Gravimetric Analysis (TGA)

TGA were carried out in TG 209 F1 Libra (Netzch-Gerätebau GmbH, Germany). Evaporation of water from gels was measured by heating ~10 mg of sample contained in alumina pans at a rate of 10.0 K.min^−1^ from 25 to 600 °C in nitrogen. Measurements were performed in triplicate.

#### Microindentation of agar substrates

The microindentation experiments were carried out as described in Ziege et al.^41^. After the agar substrate preparation, 2–3 agar plates were used for microindentation. Seven measurements were performed on each sample. The distance between two measurement points was at least 250 μm in x and y directions and the depth of the indentation was between 10 and 30 µm. A TI 950 Triboindenter (Hysitron Inc.) was used to determine the load–displacement curves after calibration of the instrument in air. Loading rates ranged from 20 to 30 μm.s^−1^, which corresponds to loading and unloading times of 10 s. The loading portion of all curves were fitted with a Hertzian contact model over an indentation range of 0 to 10 µm to obtain the reduced Young’s modulus E_r_.

### Bacterial strain and growth

The biofilm-forming bacterial strain *E. coli* K-12 W3110 was used throughout this study. Salt-free agar plates (15 mm diameter) were prepared with 1.8% w/v of bacteriological grade agar−agar (Roth, 2266), supplemented with 0.5%, 1.0%, 2.0%, 4.0% or 8.0% w/v tryptone (Roth, 8952) and 0.25%, 0.5%, 1.0%, 2.0% and 4.0% w/v yeast extract (Roth, 2363), respectively. After agar pouring, the plates were left to dry for 10 min with the lid open and 10 min with the lid partially open to avoid future condensation. Each agar plate was left to rest for 48 h at room temperature before bacteria seeding. A suspension of bacteria was prepared from a single colony and grown overnight in Luria−Bertani (LB) medium at 37 °C with shaking at 250 rpm. Each plate was inoculated with arrays of 9 drops of 5 μL of bacterial suspension (OD600 ∼ 0.5 after 10x dilution). After inoculation, the excess water evaporated from the drops and left bacteria-rich dry traces of comparable sizes from 4 to 8 mm diameter, depending on the growth condition. Biofilms were grown for 5 days in total (∼120 h) inside an incubator at 28 °C. Monitoring the relative humidity in the incubator showed that it remains around 30%RH.

### Biofilm imaging

Three biofilms per condition were imaged in bright field with a stereomicroscope (AxioZoomV.16, Zeiss, Germany) using the tiling function of the acquisition software (Zen 2.6 Blue edition, Zeiss, Germany). To estimate the biofilm size, at least 12 independent biofilms were used to calculate their area using the Fiji software^52^.

The fluorescence images were taken from biofilms grown on salt-free agar supplemented with the fluorescent dye Direct Red 23 (Pontamine Fast Scarlett 4b) (CAS 3441-14-13 Sigma-Aldrich, Germany) in a final concentration of 0.03 g/L^23,53^.

Three biofilms per condition were imaged with the stereomicroscope using the Texas Red filter set 45 (Zeiss, Germany) allowing for excitation around 560 nm ( ± 20 nm) and collection of emission between 592 and 667 nm with a beamsplitter at 585 nm. The background of each image was then subtracted using the Fiji software.

The protocol established to obtain cross sections of living biofilms was adapted from Ziege et al.^41^. Briefly, the biofilms of interest were isolated by trimming the underlying agar substrate slowly immersed with 50 °C hot liquid salt-free agar without added nutrients. The resultant agar−biofilm−agar sandwiches were then cut into ∼1 mm-thick slices using a blade, and placed in thin glass slides. The slices of each biofilm were observed with a LEICA confocal microscope SP8 FALCON (Leica, Mannheim, Germany) with an oil immersion 63X objective (1.2NA) under excitation at 552 nm and collecting signal in the emission range 600–700 nm. Images were taken and analyzed with the LAS X software. A total of 5 cross-sections per biofilm were imaged. To calculate the average intensity values of each cross-section, we used Fiji software was used^52^.

### Biofilm dry mass and water uptake

The water content and water uptake of the biofilms were determined by scrapping 7 biofilms per condition from the respective agar substrates after 5 days of growth (~120 h). Biofilms were placed in plastic weighing boats, and oven-dried at 60 °C for 3 h. Wet and dry masses (M_wet_, M_dry_) were determined before and after drying weighing the biofilm masses in an analytical balance, sensing up to 3 decimal positions^41^. To determine the water uptake (W_up_), we added Millipure water in excess (5 mL) to the biofilms harvested from each condition, covered them with aluminum foil to avoid evaporation and left overnight. The water excess was removed and the biofilm samples were weighed again (M_rewet_). The biofilms water content in each growth condition was estimated with (1)

1 $${\rm{W}}=({{\rm{M}}}_{{\rm{wet}}}\,-{{\rm{M}}}_{{\rm{dry}}})/{{\rm{M}}}_{{\rm{wet}}}\times 100 \% {\rm{w}}/{\rm{w}}$$

The percentage of water uptake of biofilms after rehydration *(%W*_*up*_) was determined with respect to the biofilm initial wet mass as described in (2)

2 $$\% {W}_{{up}}=({{\rm{M}}}_{{\rm{rewet}}}-{{\rm{M}}}_{{\rm{dry}}})/{{\rm{M}}}_{{\rm{wet}}}\times 100 \% {\rm{w}}/{\rm{w}}$$

The water uptake per gram of dry biofilm *(W*_*up*_) was calculated with (3)

3 $${W}_{{up}}=({{\rm{M}}}_{{\rm{rewet}}}-{{\rm{M}}}_{{\rm{dry}}})/{{\rm{M}}}_{{\rm{dry}}}$$

All procedures were carried out in four independent experiments.

### Microindentation on biofilms

Biofilms were grown for 5 days and stored at 4 °C, the Petri dishes were sealed with parafilm to prevent evaporation. A total of 4 biofilm samples were tested per condition. Microindentation measurements were carried out using a TI 950 Triboindenter instrument (Hysitron Inc.) to determine the load−displacement curves p−δ. The instrument was calibrated in air. Indentations were performed with a spherical diamond tip of radius R = 50 μm on a stage designed for the measurement of soft biological samples. The sample surface was approached up to 400–300 μm above the surface and retracted to the starting position while recording the measured force over the whole range. Loading rates ranged from 20 to 30 μm.s^−1^, which corresponds to loading and unloading times of 10 s. Between 8 and 10 measurements were performed in the central region of each biofilm, which were still attached to the respective agar substrates. Average and standard deviations were calculated over all measurements from a respective condition. The lateral distance between two measurement points was at least 200 μm in x and y directions. A Hertzian contact model was fitted to the loading part of the curve (indentation depth range δ = 0–10 μm) to obtain the reduced Young’s modulus (see Supplementary Fig. 3 for more details).

### Water activity measurement and osmotic gradient calculation

Four biofilms were scrapped per plate and spread in a sample container to be measured in a dew point water activity meter (WaterLab, Steroglass S.R.L, Italy). Measurements started after the chamber reached 25 °C ± 1 °C, and the final water activity value (a_w_) was obtained once thermal equilibrium is reached between the water vapor phase in the chamber and the liquid vapor phase in the sample. Measurements were done in triplicates.

The water activity of a solution can be related to the osmotic pressure (∏) by (4):

4 $$\prod =\,-\frac{{\rm{RT}}}{{{\rm{V}}}_{{\rm{w}}}}\mathrm{ln}({{\rm{a}}}_{{\rm{w}}})$$

where V_w_ is the molar volume of water in solution (V_w_ = 1.8 × 10^−5^ m³/mol), R is the universal gas constant (R = 8.314 m³.Pa/(K.mol)), and T is the absolute temperature (T = 298.15 K). We define the osmotic gradient between the biofilm and the agar (∆∏) as (5),

5 $$\Delta =-\frac{{\rm{RT}}}{{{\rm{V}}}_{{\rm{w}}}}{\mathrm{ln}}\left(\frac{{{\rm{a}}}_{{\rm{wb}}}}{{{\rm{a}}}_{{\rm{wa}}}}\right)$$

### Bacterial growth curve

Salt-free Luria-Bertani liquid media were prepared at different nutrient concentrations for this experiment (0.75, 1.5, 3.0, 6.0 or 12.0% w/v). Single colonies of E. coli W3110 K-12 bacteria were first grown on LB agar and were then inoculated in 10 mL of salt free LB media in 50 mL Falcon® tubes (one single colony per condition). The cultures were grown aerobically in a shaker (250 rpm) at 37 °C for 8 h. Bacterial growth was monitored by measuring the OD600nm of each culture every 30 min. Three independent samples were tested for each condition.

### Bacterial metabolic activity (MTT)

Salt-free Luria-Bertani liquid media were prepared containing different nutrient concentrations for this experiment (0.75, 1.5, 3.0, 6.0 or 12.0% w/v). Single colonies of E. coli W3110 K-12 bacteria were first grown on LB agar and were then inoculated in 10 mL of salt free LB media in 50 mL Falcon® tubes (one single colony per condition). The cultures were grown aerobically in a shaker (250 rpm) at 37 °C for 8 h. Aliquots of sample from the liquid cultures were taken at 60, 120, 240 and 360 min after the start point of the experiment to measure the metabolic activity of the bacteria. A protocol adapted from Oh et al.^51^ was used to perform an MTT (3-(4,5-dimethylthiazol-2-yl)-2,5-diphenyltetrazolium bromide) assay.

Briefly, 100 µL of sample were washed twice in PBS 1X by centrifugation at 1000 g for 5 min at 4 °C. The pellet was resuspended to a final volume of 100 µL PBS 1X. In a 96-well microplate, each sample was incubated with 10 µL of MTT 0.5 mg/mL in PBS for 15 min at 37 °C, protected from the light. After incubation, 100 µL of NaOH 1 M was added to each sample in order to solubilize the formazan crystals. Absorbance values were recorded at 570 nm and PBS 1X was used as blank. The experiment was done with three independent bacteria liquid cultures per condition.

### Biofilm total protein extraction

One biofilm per condition was scrapped and placed in an Epperndorf® tube with 200 µL of lysis buffer (1% SDS, 1% RIPA, 1% Tritonx100). The biofilms were disrupted mechanically using a XENOX MHX 68500 homogenizer for one minute, before being sonicated for 15 min in a bath sonicator. After sonication, biofilms were again disrupted for one minute and incubated at 26 °C for 20 min. The samples were then centrifuge at 17,000 g for 10 min at 4 °C and the pellet was discarded. Aliquots of 50 µL were taken and mixed with an equal volume of an acetone/methanol 1:1 solution for protein precipitation. After an overnight incubation at −20 °C, the samples were centrifuged at 18,000 g for 30 min at 4 °C. The supernatant of each sample was discarded and pellets were resuspended in 15 µL of PBS 1X for protein quantification.

Protein quantification was done using a Bradford assay. Briefly, in a 96-well microplate, 5 µL of sample was incubated with 195 µL of Bradford reagent (Quick Start™ Bradford 1x Dye Reagent #5000205, BioRad). Absorbance was measured at 550 nm after a 5–10 min incubation protected from the light. BSA (heat shock fraction, protease free, fatty acid free, essentially globulin free, pH 7, ≥ 98%, (A7030) Sigma) was used in different concentrations as a calibration curve. The experiments were repeated with three independent biofilms.

### Curli fiber purification

Fiber purification involved a similar process as reported in previous works^16,54^. Briefly, a total of 27 biofilms (~1 g of biofilm material) were scraped from the surface of the substrates. Biofilms were blended five times on ice with an XENOX MHX 68500 homogenizer for 1 min at 2-min intervals. The bacteria were pelleted by centrifuging two times at low speed (5000 g at 4 °C for 10 min). A final concentration of NaCl 150 mM was added to the supernatant and the curli pelleted by centrifuging at 12,000 g at 4 °C for 10 min. The pellet was resuspended in 1 mL of solution containing 10 mM tris (pH 7.4) and 150 mM NaCl, and incubated on ice for 30 min before being centrifuged at 16,000 g at 4 °C for 10 min. This washing procedure was repeated thrice. The pellet was then resuspended in 1 mL of 10 mM tris solution (pH 7.4) and pelleted as described above (16,000 g at 4 °C for 10 min). The pellet was again suspended in 1 mL of 10 mM tris (pH 7.4) and centrifuged at 17,000 g at 4 °C for 10 min. This washing step was repeated twice. The pellet was then resuspended in 1 mL of SDS 1% v/v solution and incubated for 30 min. The fibers were pelleted by centrifuging at 19,000 g at 4 °C for 15 min. The pellet was resuspended in 1 mL of Milli-Q water. This washing procedure was repeated thrice. The last resuspension was done in 0.1 mL of Milli-Q water supplemented with 0.02% sodium azide. The fiber suspension was stored at 4 °C for later use. The protein concentration in monomeric units of the suspensions was determined by the absorbance from an aliquot incubated in 8 M urea at 25 °C for 2 h, a treatment leading to complete dissociation of the fibrils as verified by Thioflavin T measurements (no signal from the fibers after treatment with 8 M urea). To account for fiber polymorphism, all experiments were performed on each batch of purified fibers. Each replicate of experiment represents an independent purification process.

### Curli fiber identification: SDS-PAGE

The purified fibers were pelleted for 20 min at 4 °C and 19,000 g. Each pellet was treated with 20 µL of formic acid. After evaporating the acid, an SDS-PAGE 15% acrylamide was carried out. For visualizing the protein bands, READYBLUE® PROTEIN GEL STAIN (Sigma-Aldrich, Germany) was used.

A second SDS-PAGE 15% acrylamide was run for a Western Blot using anti-CsgA as primary antibody (Catalog No. ABIN7141612), and Anti-IgG rabbit (HRP) (Catalog No. ABIN2690388) as secondary antibody (Antibodies-Online). Bands in the Western Blot were reveled using a chemiluminescent enhancer reagent for HRP (Pierce™ ECL Western Blotting-Substrate, ThermoFisher, Germany).

### Curli fiber identification: Congo Red UV-visible spectroscopy

A total of 5 µL of purified fibers from each condition was incubated with a 0.1 mg/mL congo red solution (Sigma-Aldrich, Germany) for 15 min at room temperature and shaking. After incubation, the samples were centrifuged for 10 min at 19 500 g and 4 °C to remove the unbound congo red (supernatant); the pellet containing purified curli fibers stained with congo red were resuspended in miliQ water. A UV-VIS Spicord210 Plus (Analytik Jena GmbH+Co) spectrometer was used to record the spectra of the different samples. Two different absorbance range were used; from 240 to 300 nm for protein absorbance (fibers), and from 325 to 650 nm for congo red absorption. Corrected steady-state emission spectra were acquired with a FluoroMax®-4 spectrofluorometer (HORIBA). Spectra were recorded at 25 °C using a 3-mm path cuvette (Hellma® Analytics). The CR samples were measured using λ_exc_ = 540 nm and spectral bandwidths of 10 nm^55^.

### Transmission electron microscopy (TEM)

2 μL drops of fiber suspension were adsorbed onto Formvar-coated carbon grids (200 mesh), washed with Milli-Q water, and stained with 1% w/v uranyl acetate. The samples were imaged in a JEOL-ARM F200 transmission electron microscope (JEOL GmbH, Germany) equipped with two correctors for imaging and probing. For the observations, we applied an acceleration voltage of 200 kV.

### Attenuated total reflectance Fourier transform infrared spectroscopy (ATR-FTIR)

IR spectra were acquired with a spectrophotometer (Vertex 70 v, Bruker Optik GmbH, Germany) equipped with a single reflection diamond reflectance accessory continuously purged with dry air to reduce water vapor distortions in the spectra. Fibers samples in Milli-Q water (∼10 μL) were spread on a diamond crystal surface, dried under N_2_ flow to obtain the protein spectra. A total of 64 accumulations were recorded at 25 °C using a nominal resolution of 4 cm^−1^.

Spectra were processed using Kinetic software developed by Dr. Erik Goormaghtigh at the Structure and Function of Membrane Biology Laboratory, Université Libre de Bruxelles, Brussels, Belgium. After subtraction of water vapor and side chain contributions, the spectra were baseline corrected and area normalized between 1700 and 1600 cm^−1^ (Supplementary Fig. 11). For a better visualization of the overlapping components arising from the distinct structural elements, the spectra were deconvoluted using Lorentzian deconvolution factor with a full width at the half maximum (FWHM) of 20 cm^−1^ and a Gaussian apodization factor with a FWHM of 30 cm^−1^ to achieve a line narrowing factor K = 1.5^28^. Second derivative was performed on the Fourier self-deconvoluted spectra for band assignment. The bands identified by both procedures were used as initial parameters for a least square iterative curve fitting of the original IR band (K = 1) in the amide I’ region, using mixed Gaussian/Lorentzian bands. Peak positions of each identified individual component were constrained within ±2 cm^−1^ of the initial value (Table 2).

**Table 2:** FWHH input values in cm^−1^ and their physically plausible ranges expected for each type of secondary structure^18,27,28^

Secondary structure component		FWHH input (cm^−1^)	Lower limit (cm^−1^)	Upper limit (cm^−1^)
β-sheet	High wavenumber component	9	8	11
	Low wavenumber component	17	14	19
α-helix/ random		20	5	30
Turns		20	5	30

### Circular dichroism spectroscopy

Spectra of 5 µM CsgA monomer concentration of fiber solution in Milli-Q water were recorded with a Chirascan CD spectrometer (Applied Photophysics, Leatherhead, Surrey, UK). A quartz cuvette with 1 mm path length (Hellma, Müllheim, Germany) was used. Spectra are acquired between 190 nm and 250 nm wavelengths, with 1 nm step size, 1 nm band-width and 0.7 s integration time per point. Milli-Q water was used to define the measurement background, which was automatically subtracted during acquisition. The experiments were repeated thrice for each condition. Each measurement was an average of three scans.

### Fluorescence spectroscopy

Corrected steady-state emission spectra were acquired with a FluoroMax®-4 spectrofluorometer (HORIBA). Spectra were recorded at 25 °C using a 3-mm path cuvette (Hellma® Analytics). Thioflavin T(ThioT) measurements were performed at final concentrations of 3 μM protein, 1 mM probe in Glycine buffer, pH 8.2, using λ_exc_ = 446 nm and spectral bandwidths of 10 nm. Nile red (NR) measurements were performed at a final concentration of 7 μM protein, 7 mM probe in water, using λ_exc_ = 560 nm and spectral bandwidths of 10 nm. Intrinsic fluorescence spectra (5 μM protein) were acquired using λ_exc_ = 280 nm and 5/5 nm slit bandwidths. 5 µM solution of soluble Trp (N-Acetyl-L-tryptophan, NATA) was included as a reference of the emission spectrum of a fully exposed Trp.

Fiber polarity is defined as the ratio between the position of the emission maximum of the nile red (NR) in buffer and the position of the emission maximum of the NR bound to each fiber (6):

6 $${Fiber\; polarity}=\frac{{\rm{Position\; of\; emission\; maximum}}{{NR}}_{{fiber}}\,({\rm{nm}})}{{\rm{Position\; of\; emission\; maximum}}{{NR}}_{{buffer}}\,({\rm{nm}})}$$

A polarity value of 1.00 indicates highly polar fibers.

Trp hydrophobicity is defined as the ratio between the position of the emission maximum of the Trp population in the fibers and the position of the emission maximum of soluble Trp (NATA) in buffer (as reference for the position of the emission maximum when we have the highest exposure to the solvent) (7):

7 $${Trp\; hydrophobicity}=\frac{{\rm{Position\; of\; emission\; maximum}}{{Trp}}_{{fiber}}\,({\rm{nm}})}{{\rm{Position\; of\; emission\; maximum}}{{NATA}}_{{buffer}}\,({\rm{nm}})}$$

A hydrophobicity value of 1.00 indicates more exposure of the Trp to the solvent.

### Chemical stability assay

To test the chemical stability of the protein fibers, 5 μM samples were prepared by incubation of increasing urea concentration (0–8 M) and left for 2 h at room temperature to ensure equilibrium. ThioT was then added in a final concentration of 1 mM and fluorescence emission spectra of the samples were acquired under excitation at λ_exc_ = 446 nm and spectral bandwidth of 5 nm. Emission of the ThioT in urea was done and no significant signal was detected.

The IC50 value was estimated by fitting the data to a linear regression (Y = a * X + b) and then calculating IC50 = (0.5 - b)/a.

### Purified curli fiber denaturation and CsgA polymerization

Samples of purified curli fibers were precipitated by centrifugation for 25 min at 18,000 g at 4 °C. In order to denature the purified curli fibers, 20 µL of formic acid (FA) at 98% was added to each sample and left to evaporate after 15 min^48^. After denaturation, 60 µL of milliQ water was added to the CsgA monomers and the samples were immediately prepared for polymerization in 96-well microplates (Supplementary Fig. 16).

Each well contained a final volume of 50 µL made of 10 µL of CsgA monomer solution, 5 µL of ThioT 100 µM and 35 µL of solvent. The solvent was either Tris-HCl 10 mM NaCl 50 mM, LB medium containing 1.5% w/v, or LB medium containing 6.0% w/v. The fibrillation of CsgA was monitored by placing the microplate in a microplate reader (BioTek Cytation5, Agilent), where the ThioT emission was acquired every 10 min for 10 h. The temperature was set at 25 °C and the plate was shaken prior to every measurement. The fibrillation process was performed and studied 4 times for each condition.

The CsgA monomers from the denatured samples were identified with 15% acrylamide SDS-PAGE electrophoresis gel, and CD spectroscopy. The polymerized CsgA fibers were identified using ATR-FTIR spectroscopy, transmission electronic (TE) microscopy, and ThioT fluorescence emission. These measurements were done twice.

### Statistical analysis

For each experiment, 3 to 4 fiber solutions were used, where each solution came from different fiber purification batches. For each purification, 27 biofilms were cultured in each of the 5 growth conditions tested (i.e., on salt-free LB-agar plate containing 0.75%, 1.5%, 3.0%, 6.0% and 12.0% w/v nutrient, respectively). For each batch of biofilm culture, the different samples of fibers obtained were treated simultaneously (or in consecutive days) to prevent variability due to unavoidable slight variations in the implementation of the protocols (e.g., temperature and humidity in the laboratory during agar preparation and/or biofilm seeding). For statistical analysis, a One-way ANOVA test was carried out. Mechanical properties data was analyzed using a Mann–Whitney *U* test. Unless otherwise stated in the caption, Dunn’s post-test for multiple comparisons were done with respect to the 1.5% w/v nutrient salt-free LB-agar condition, considered as the standard seeding condition. Details of each test are described in the legend of the figures.

## Supplementary information

Supplementary information

## Data Availability

The data that support the findings of this study are available from the corresponding authors upon reasonable request.
