# Supplementary material for: Nutrient availability influences E. coli biofilm properties and the structure of purified curli amyloid fibers
Source: NPJ Biofilms Microbiomes. 2024 Dec 4;10:143. doi: 10.1038/s41522-024-00619-0 (PMC11618413; doi:10.1038/s41522-024-00619-0)
Supplement: Supplementary file 1 — Supplementary information [file 41522_2024_619_MOESM1_ESM.pdf]

Nutrient availability influences *E. coli* biofilm properties and the structure of purified curli amyloid fibers

Macarena Siri*[1,2], Mónica Vázquez-Dávila[1], Carolina Sotelo Guzman[1], Cécile M. Bidan*[1]

1. *Max Planck Institute of Colloids and Interfaces, Department of Biomaterials, Potsdam14476, Germany*
2. *Max Planck Queensland Centre, Potsdam 14476, Germany*

**corresponding authors*

Supporting Information

Salt-free agar characterization	Supplementary Figure 1
Biofilm morphology	Supplementary Figure 2
Biofilm matrix intensity (Direct Red 23)	Supplementary Figure 3
	Supplementary Figure 4
Biofilm mass	Supplementary Table1
	Supplementary Figure 5
Microindentation loading curve	Supplementary Figure 6
Biofilm rehydration	Supplementary Figure 7
Biofilm total protein extraction	Supplementary Figure 8
Bacteria in liquid media	Supplementary Figure 9
	Supplementary Figure 10
Purified curli fiber TEM images	Supplementary Figure 11
Identification of purified curli amyloid fibers	Supplementary Figure 12
	Supplementary Figure 13
ATR- FTIR spectra of the purified curli fibers	Supplementary Table 2
	Supplementary Figure 14
Fiber structure by circular dichroism (CD) spectrometry	Supplementary Figure 15
Fiber polarity	Supplementary Figure 16
Curli fibers and the csgA subunit	Supplementary Figure 17
Structure/function relationship between purified curli fibers and biofilm mechanical properties	Supplementary Figure 18
Proof of concept: from purified curli fibers to CsgA to fibers again	Supplementary Figures 19

1. Salt-free agar characterization

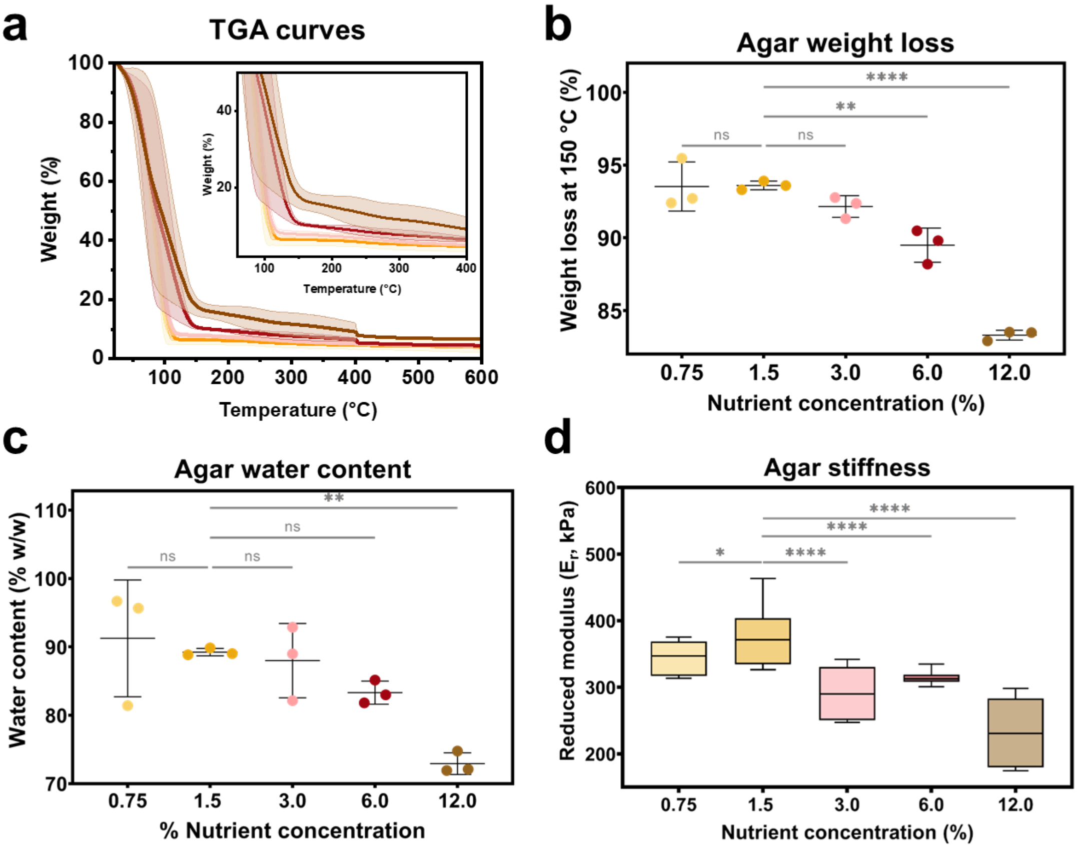

Supplementary Figure 1 Agar substrate properties. (a) Average Thermal Gravimetric Analysis (TGA) curves of each agar substrate between 90 and 600 °C. Inset is a zoom between 60 – 400 °C. N = 3. (b) Percentage of weight loss representing water loss at 150 °C for each agar substrate. (c) Water content of the agar substrates. N=3. The statistical analysis was done with One-way ANOVA (p<0.0001, **** | p<0.001, *** | p<0.01, ** | p<0.05, * | ns = non-significant), where the 1.5 % w/v nutrient concentration condition was used as reference for the post-test multicomparisons. (d) Mechanical properties (stiffness) of the different agar substrates studied. N=14 microindentation curves of 2-3 agar substrates per condition. The number of individual measurements is n = 10–23 per condition. The statistical analysis was done with Man Whitney U test (p<0.00=1, **** |p<0.001, *** | p<0.01, ** | p<0.05, * | ns = non-significant), where the 1.5 % nutrient concentration condition was used as reference for the post-test multicomparisons.

We estimated the substrates (agar) free water content in every condition by carrying out thermal gravimetric analysis (TGA) (**Supplementary Figure 1a**). We tested the behavior of the substrates from 25 to 600 °C. The results show that the higher the nutrient concentration in the agar substrate, the lower content of free water is in the substrate (**Supplementary Figure 1a**). The loss of weakly linked water (Van der Waal forces) is indicated by a sharp loss of mass between 100 and 150 °C depending of the substrate. For substrates with low nutrient concentration, a lower temperature is needed to release weakly linked water compared to high nutrient concentration substrates. The total weight loss at 150 °C is correspond to the mass free water released (**Supplementary Figure 1b**).[1] Substrates with low nutrient concentration (0.75, 1.5 and 3.0 % w/v) lose ~90 % of their weight, while substrates with high nutrient concentration

(6.0 and 12.0 % w/v) lose 83 and 73 % of their weight respectively. We also analyzed the water content of the salt-free LB-agar substrates by dehydration (**Supplementary Figure 1c**). Low nutrient concentration substrates (0.75 and 1.5 % w/v) have 91 and 89 w/w % of water, respectively. Higher nutrient concentration substrates (3.0, 6.0 and 12.0 % w/v) have 87, 83 and 73 % w/w of water.

Analyzing the mechanical properties of the agar substrates revealed that the higher their nutrient content, the lower their stiffness (**Supplementary Figure 1d-e**).

2. Biofilm morphology

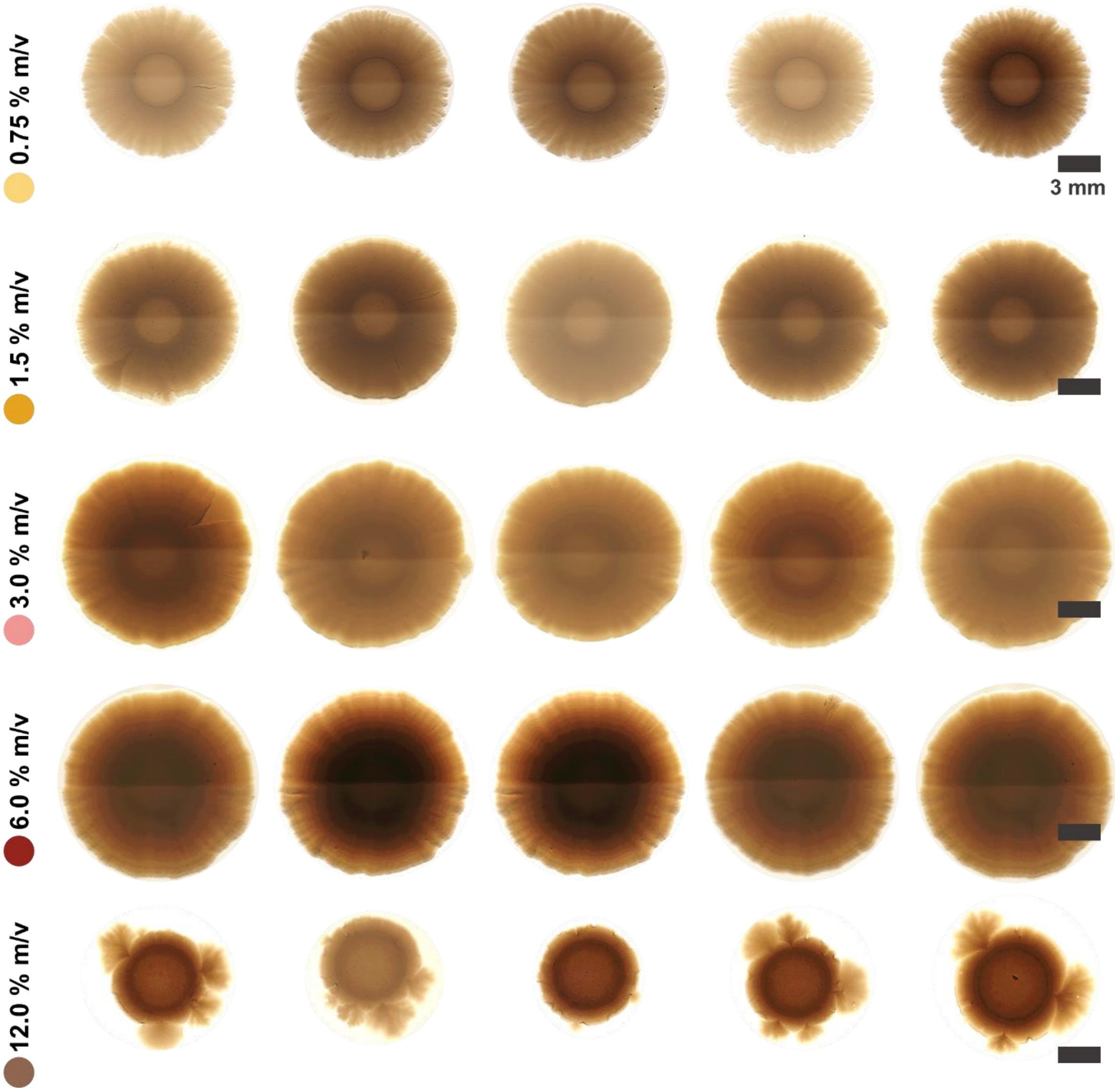

Supplementary Figure 2 Images from biofilms grown at different substrate nutrient concentrations. The images are representative of the different morphologies. Scale bar = 3 mm.

3. Biofilm matrix staining with Direct Red 23

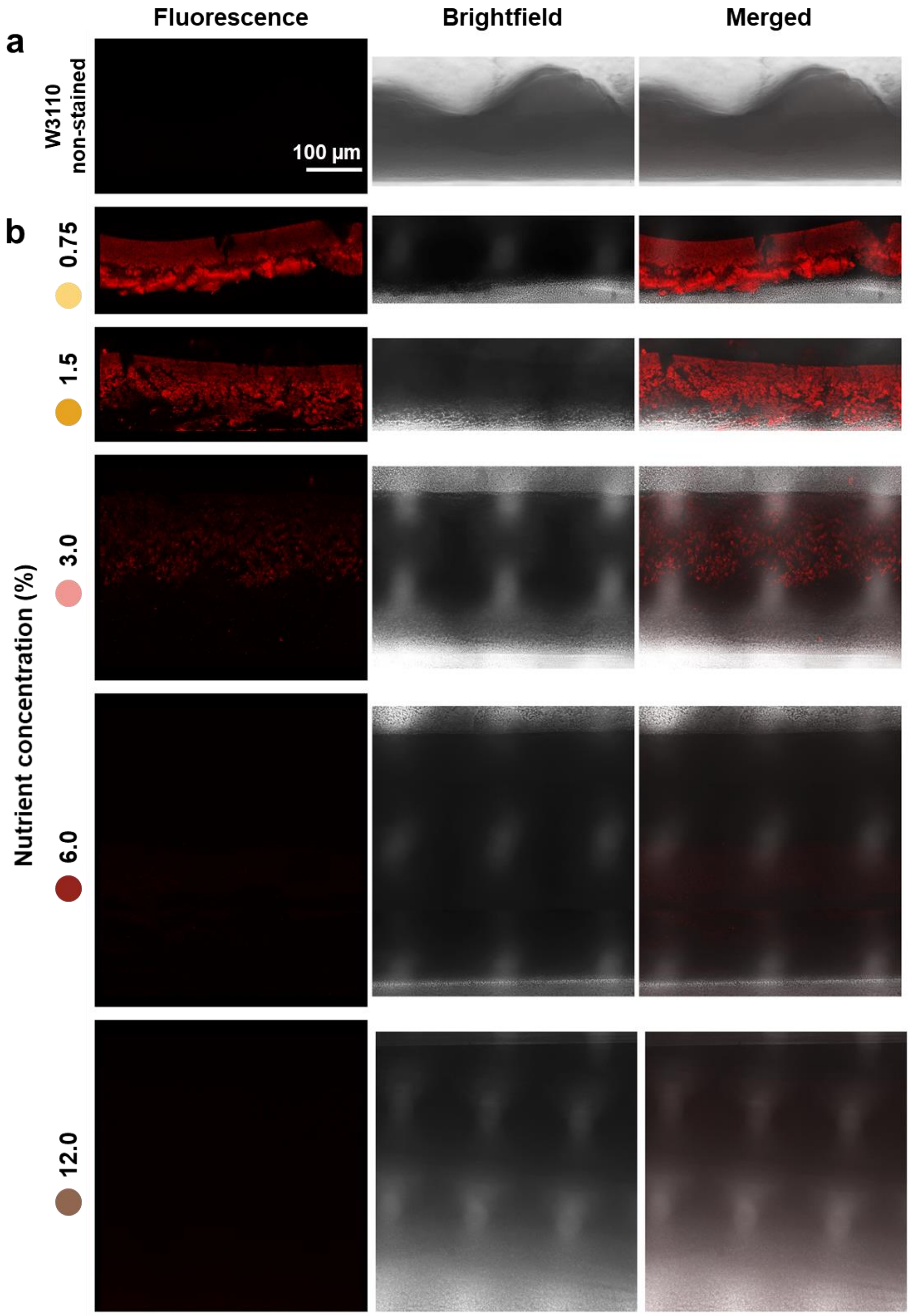

Supplementary Figure 3 Cross-section of the biofilms obtained in different growth conditions. Cross-section of *E. coli* W3110 biofilms depicting fluorescence, brightfield and merged images. (a) The first row depicts the cross-section of non-stained *E. coli* W3110 biofilm grown on salt-free LB agar containing 1.5 %w/v nutrients. (b) Cross-sections of *E. coli* W3110 biofilms stained with Direct Red 23 grown on salt-free LB agar in increasing nutrient concentration. The illumination pattern observed in the Brightfield and Merged column are due to the stitching process. The limit

between agar substrate and biofilm of the 12 %w/v nutrient concentration condition is difficult to identify due to smearing of the biofilm when preparing the cross-section.

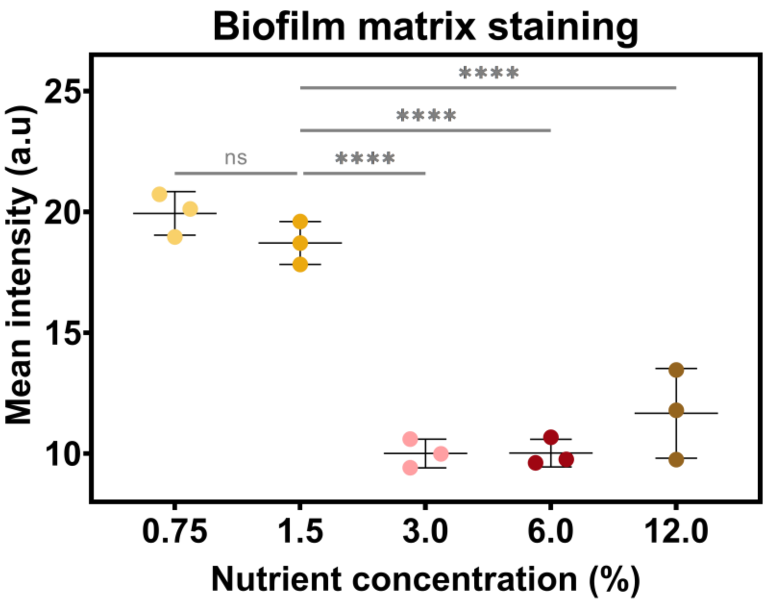

Supplementary Figure 4 Intensity of the Direct Red fluorescent staining on biofilm cross-sections. The mean intensity of the raw images of each cross-section correspond to the mean value of their histogram measured using the Fiji software.[2]

4. Biofilm mass

We considered the *E. coli* W3110 biofilms wet mass (M_{wet}) as the sum of the masses of i) the water, and ii) the bacteria and matrix, also considered as the dry mass (M_{dry}). Within M_{dry}, we discriminate between i) curli fibers (as the main matrix component) and ii) bacteria and other secondary matrix components.

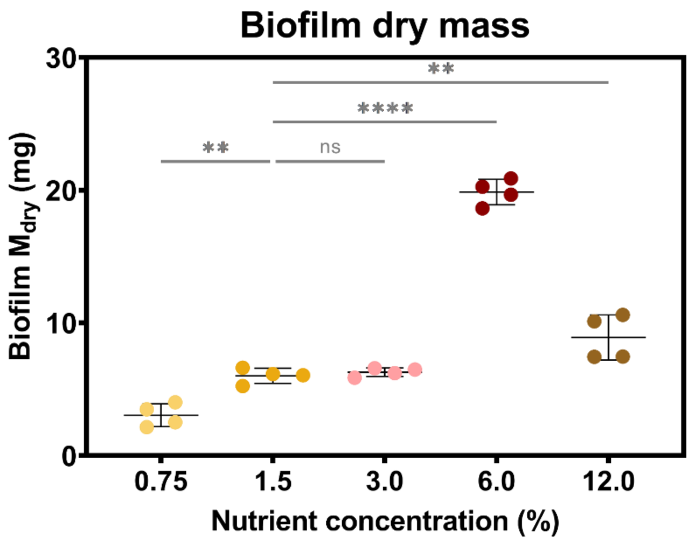

Supplementary Figure 5 Biofilm dry mass. Values correspond to the mass of a single biofilm calculated from four independent experiments. The statistical analysis was done with One-way ANOVA (p<0.0001, **** | p<0.001, *** | p<0.01, ** | p<0.05, * | ns = non-significant), where the 1.5 % w/v nutrient concentration condition was used as reference for the post-test multicomparisons.

Supplementary Table 1 Composition of the *E. coli* W3110 biofilms grown on substrates of different nutrient concentration. The total mass corresponds to the sum of the water content and the dry mass. [a]The bacteria mass was estimated by subtracting the curli mass given by the CsgA quantification after purification from the dry mass. The percentages of bacteria and CsgA are given with respect to the dry mass. N= 4 biofilms per experiment.

Substrate nutrient concentration (% w/v)	Biofilm size (mm)	Units	Total mass (M_{wet})	Water	Dry mass	CsgA[a]	Bacteria[a]
0.75	17 ± 1	mg	11 ± 3	8 ± 2	3.0 ± 0.9	0.01 ± 0.00	3.0 ± 0.9
		%	100	73	27	0.35	99.65
1.50	18 ± 2	mg	24 ± 2	18 ± 2	6.0 ± 0.6	0.02 ± 0.01	6.0 ± 0.6
		%	100	75	26	0.36	99.64
3.00	20 ± 2	mg	25 ± 1	18 ± 1	6.3 ± 0.3	0.03 ± 0.01	6.3 ± 0.3
		%	100	75	26	0.50	99.50
6.00	20 ± 2	mg	60 ± 5	40 ± 4	20 ± 1	0.05 ± 0.01	20 ± 1
		%	100	67	33	0.28	99.72
12.00	13 ± 1	mg	26 ± 5	18 ± 3	9 ± 2	0.01 ± 0.00	9 ± 2
		%	100	66	34	0.1	99.9

5. Microindentation loading curves

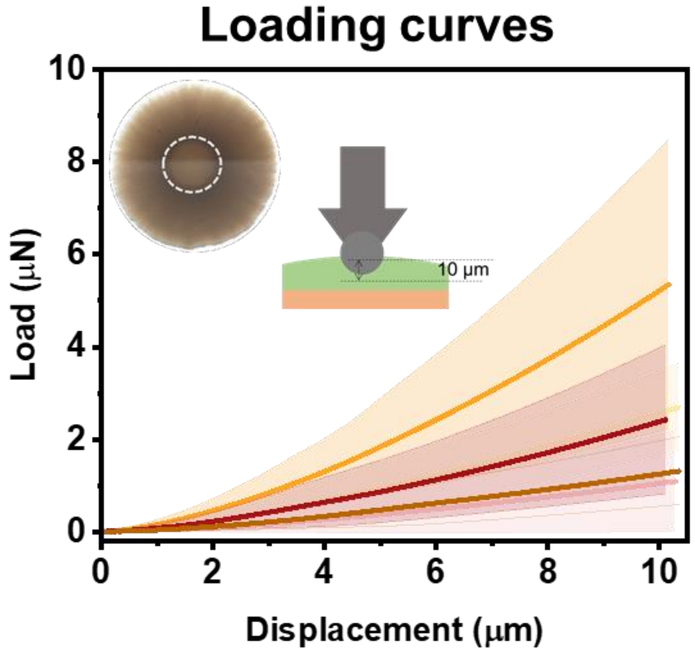

Supplementary Figure 6 Loading curves obtained from nanoindentation experiments on *E. coli* W3110 biofilms with different nutrient concentrations. Representative load−displacement curves when indenting the biofilm surface (loading curve). N= 40. Color code: 0.75 % w/v nutrient concentration (yellow), 1.50 % w/v nutrient concentration (orange), 3.00 % w/v nutrient concentration (pink), 6.00 % w/v nutrient concentration (red) and 12.0 % w/v nutrient concentration (brown). In the inset, a scheme depicts the measurement: a spherical tip (50 µm) was indented in ~10 µm of the biofilm to avoid contribution of the underlying salt-free LB agar. For more details see the experimental section.

6. Biofilm rehydration

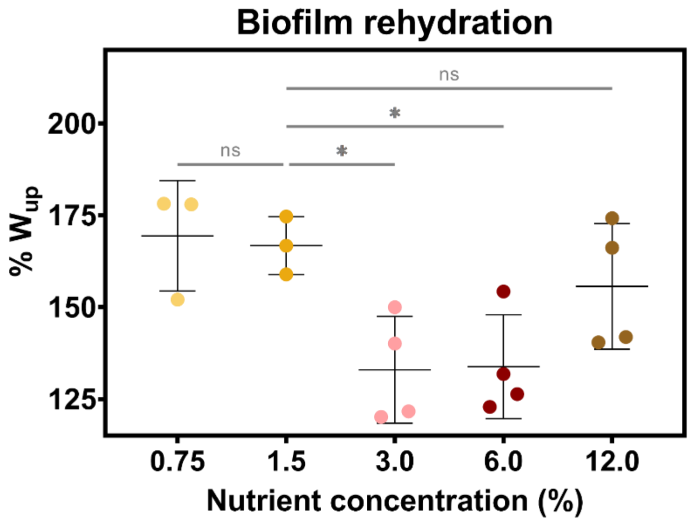

Supplementary Figure 7 Biofilm water uptake upon overnight rehydration of dry mass. The percentage of water uptake of biofilms after rehydration ($\%W_{up}$) was determined with respect to the biofilm initial wet mass: $\%W_{up} = (M_{rewet} - M_{dry})/M_{wet} \times 100$ % w/w. M_{wet}, and M_{dry} stand for wet and dry masses, respectively, and M_{rewet} stand for the biofilm weighed mass after rehydration.

7. Biofilm total protein extraction

Biofilms grown on salt-free LB agar containing 3.0 and 6.0 % w/v nutrients had the highest total protein concentration (**Supplementary Figure 3**). The trend observed for the biofilm protein concentration is similar to the one observed for the size and wet mass of the biofilms, as well as for the dry mass of the biofilms. The SDS-PAGE electrophoresis gel run with aliquots of each total protein extraction showed that the pattern of proteins expressed by the bacteria is similar between conditions.

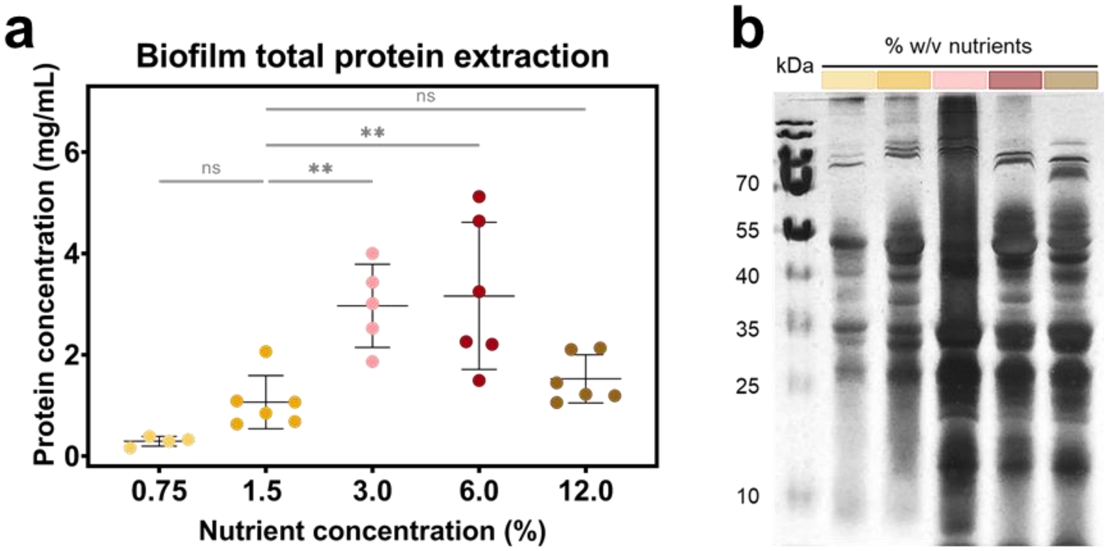

Supplementary Figure 8 (a) Biofilm total protein concentration (see experimental section for details). (b) SDS-PAGE electrophoresis gel with showing the protein pattern of the samples studied in (a).

8. Bacteria in liquid media

Growth curves allow studying the kinetics of bacterial growth in specific media. Typically, these curves present the four phases of life for bacterial culture: lag, exponential, stationary, and death (**Supplementary Figure 9**).[3] In this work, the focus was set on the exponential phase, which is the phase where the bacteria have the most active metabolism.[4]

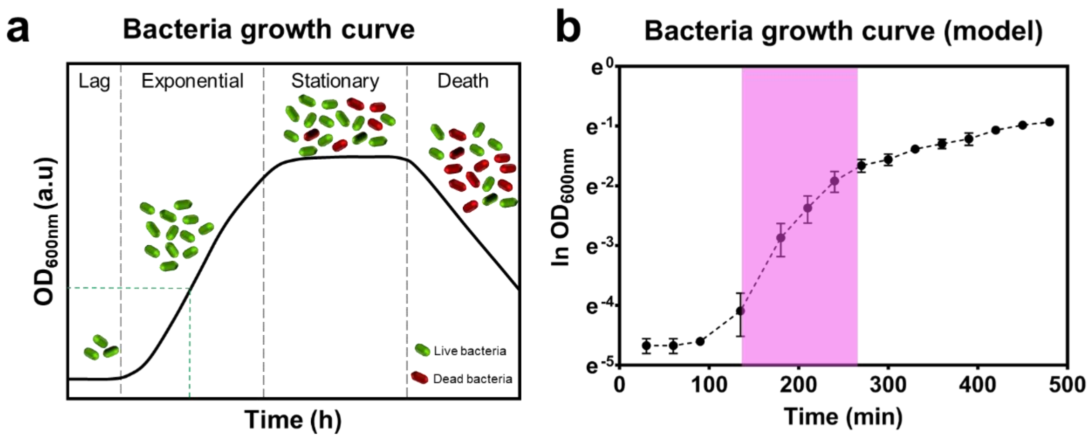

Supplementary Figure 9 Bacterial growth curve. (a) Bacteria grown in batch culture progress through four phases of growth: lag, exponential, stationary, and death. (b) Plotted growth curve ln(2) OD_{600nm} vs Time (min). Highlighted in pink is the linear section of the exponential phase.

In order to study whether the differences of protein production are due to the growth and metabolism of the bacteria, bacterial growth was monitored for 8 hours in liquid salt-free LB media (**Supplementary Figure 9**).

According to the bacterial growth curves (**Supplementary Figure 10a**), the differences in the optical density were observed at the endpoint of the experiment, which directly correlates to the number of bacteria in the suspension (**Supplementary Figure 10b**). The results suggest that the higher the nutrient concentration of the media, the higher the bacteria proliferation. Because the endpoint of our experiment was still at an early stationary phase (**Supplementary Figure 9a, Supplementary Figure 10a**), the optical density values acquired can be related to a majority of live bacteria. The bacteria metabolic activity was also studied in each phase of their growth curve (**Supplementary Figure 10c**). *E. coli* bacteria in liquid salt-free LB media containing 12.0 % w/v nutrients were more metabolically active from the beginning of the exponential phase compared to the other nutrient concentrations tested.

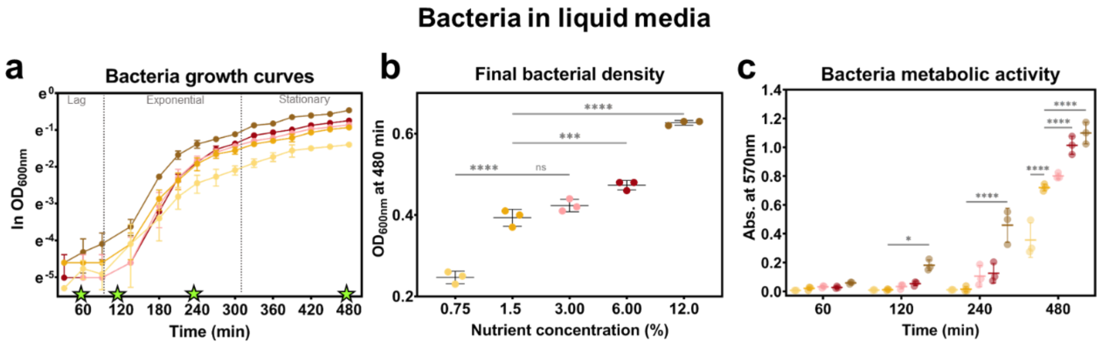

Supplementary Figure 10 Bacteria in liquid media. (a) 8-hour bacterial growth curves for each condition tested. (b) Final optical density values acquired from each condition at the endpoint of the experiment. The optical density refers to the number of bacteria produced in each solution. (c) Bacteria metabolic activity in liquid media measured using MTT at the times highlighted by green stars in panel a. All data presented here come from N = 3 independent biofilm cultures for each condition tested. The statistical analysis was done with One-way ANOVA (p<0.0001, **** | p<0.001, *** | p<0.01, ** | p<0.05, * | ns = non-significant), where the 1.5 % w/v nutrient concentration condition was used as reference

9. TEM of the purified fibers

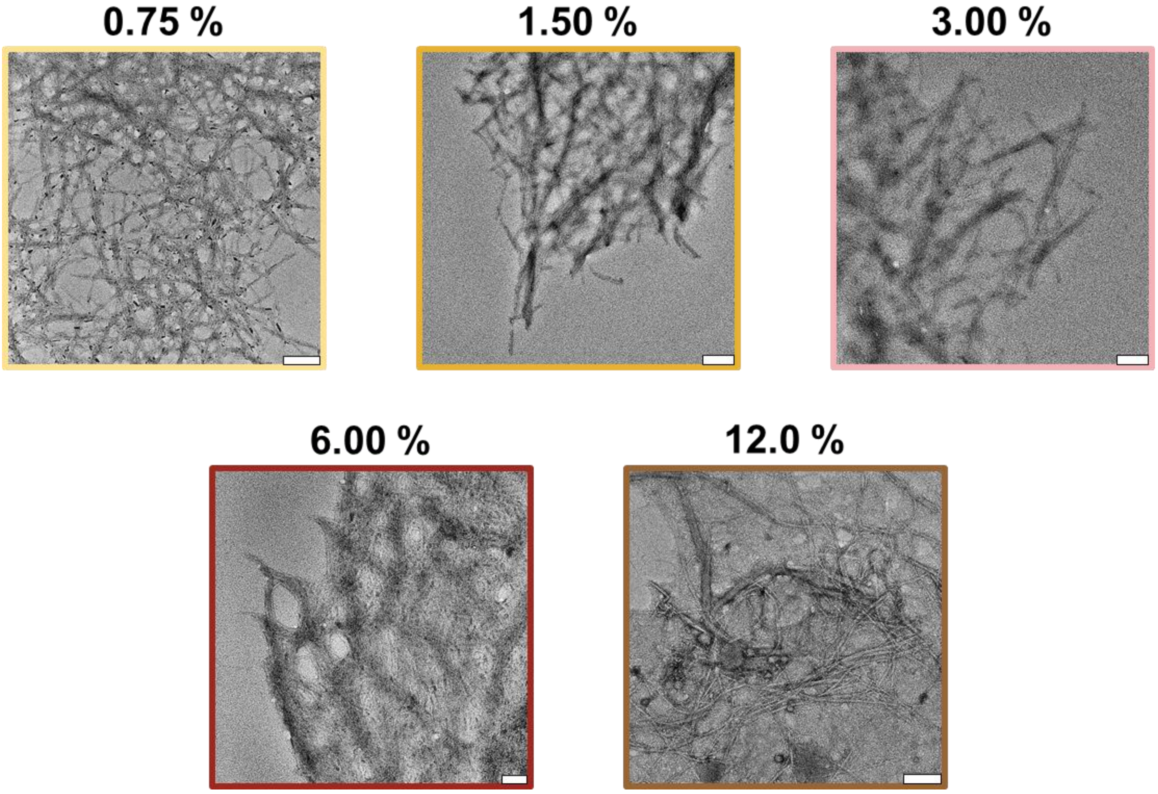

Supplementary Figure 11 Transmission electronic microscopy images of the purified fibers. Scale bar = 100 nm. Data come from 4-5 independent biofilm cultures for each condition tested.

10. Identification of purified curli amyloid fibers

a. Electrophoresis SDS-PAGE

After the fiber purification process, the samples were treated with formic acid 100 % to depolymerize the fibers to their monomer CsgA. SDS-PAGE allowed us to identify bands at ~17 and ~34 kDa, molecular weight of the CsgA monomer and dimer, respectively (**Supplementary Figure 12a**). These bands were then further recognized by antibodies to CsgA in a Western Blot (**Supplementary Figure 12b**).[5] The different intensities observed form the bands in the Western Blot matches the observations from the fiber yield quantification (**Figure 2d**).

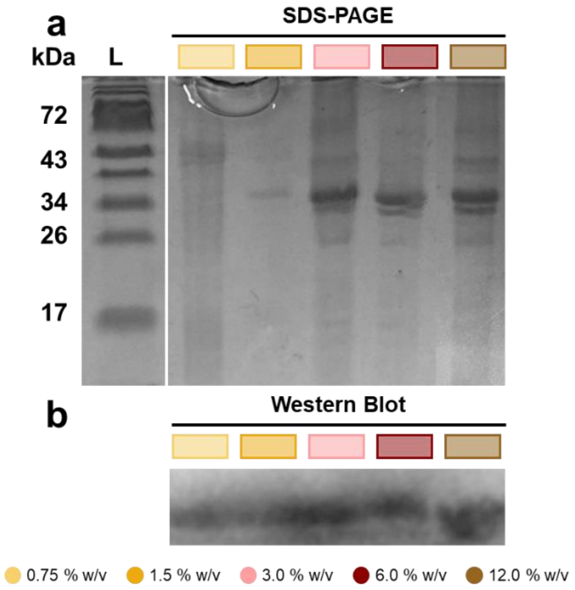

Supplementary Figure 12 Identification of CsgA. (a) Electrophoresis SDS-PAGE gel. Molecular weight marker (L) was added to help identify the molecular weight of the bands in the different lanes showing CsgA dimer (34 kDa) after treating the extracted curli amyloid fibers from biofilms with formic acid. (b) Western Blots of samples in (a) stained with Anti-CsgA as primary antibody and Anti-Rabbit IgG as secondary antibody.

b. Binding to congo red

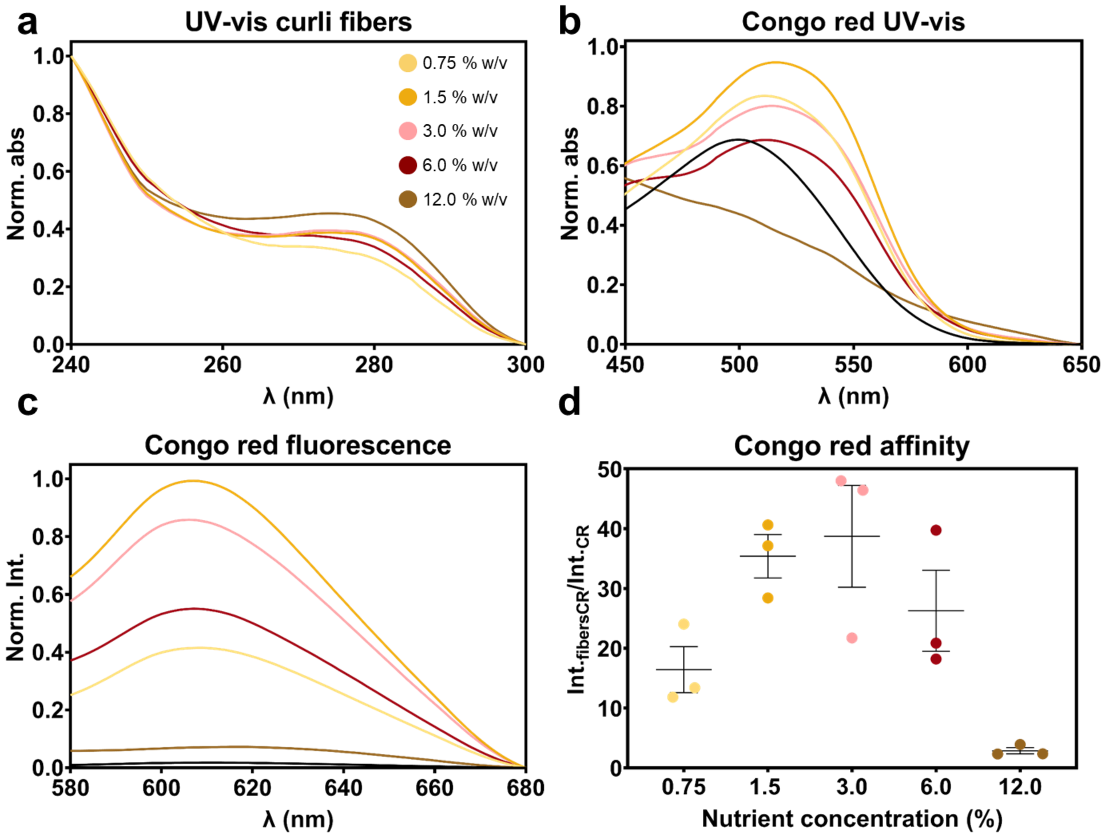

Supplementary Figure 13 Identification of purified curli amyloid fibers with congo red (CR). (a) UV-visible spectra of the purified curli amyloid fibers (λ_{abs}= 280 nm). (b) UV-visible spectra of the binding of CR to the different samples of curli amyloid fibers. Free CR spectrum is indicated in black. (c) Fluorescence emission spectra of the curli fibers bound to CR, free CR spectrum is indicated in black (λ_{exc}= 540 nm). (d) Values describing the increase in intensity of CR when bound to the purified fibers. Quantification of the increase was estimated by division of the area under the curve of each spectra of the probe with each fiber by the area under the curve of the emission spectra of the CR alone (values plotted indicating mean with SEM).

Congo red (CR) is a common dye to identify the presence of amyloid fibers. Although it is not clear how CR bounds to the amyloid fibers, two binding modes have been proposed.[6] The CR could interact along the fiber axis with the β-sheet by electrostatic or hydrophobic interactions, or the CR could intercalate in parallel between two β-strands.[6]

The purified curli fibers from the different biofilms were first identified with this dye (**Supplementary Figure 13**). The presence of fibers was assessed by protein absorbance (Abs_{280nm}) after the purification process (**Supplementary Figure 13a**). With the exception of the fibers grown in biofilms with the highest nutrient availability, all samples presented a red-shift in the CR absorbance spectrum signifying the binding between CR and fibers (**Supplementary Figure 13b**).[7]

To further study the behavior of the fibers grown on high nutrient availability (12.0 % w/v nutrient concentration) fluorescence studies were performed (**Supplementary Figure 13c-d**).

When bound to the fibers, the congo red emission spectra exhibit an increase in its intensity after an excitation at λ_{exc}= 540 nm (**Supplementary Figure 13c**). The intensity values of the different spectra varied depending on the structure of the fibers bound to CR. When normalizing the intensity value against the free CR signal, fibers produced on substrates containing 1.5 and 3.0 % w/v of nutrient showed an increase of c.a. 30 - 40 times of the CR intensity, while fibers produced on substrates containing 0.75 and 6.0 % w/v of nutrient showed an increase of c.a. 10 times of the CR intensity (**Supplementary Figure 13d**). The differences in these values among the different samples suggest changes in the structure of the purified fibers. The trend observed in this experiment, follows the trend observed for the binding of ThioT with the fibers (**Figure 3a-b**).

11. FTIR-spectra of purified curli fibers

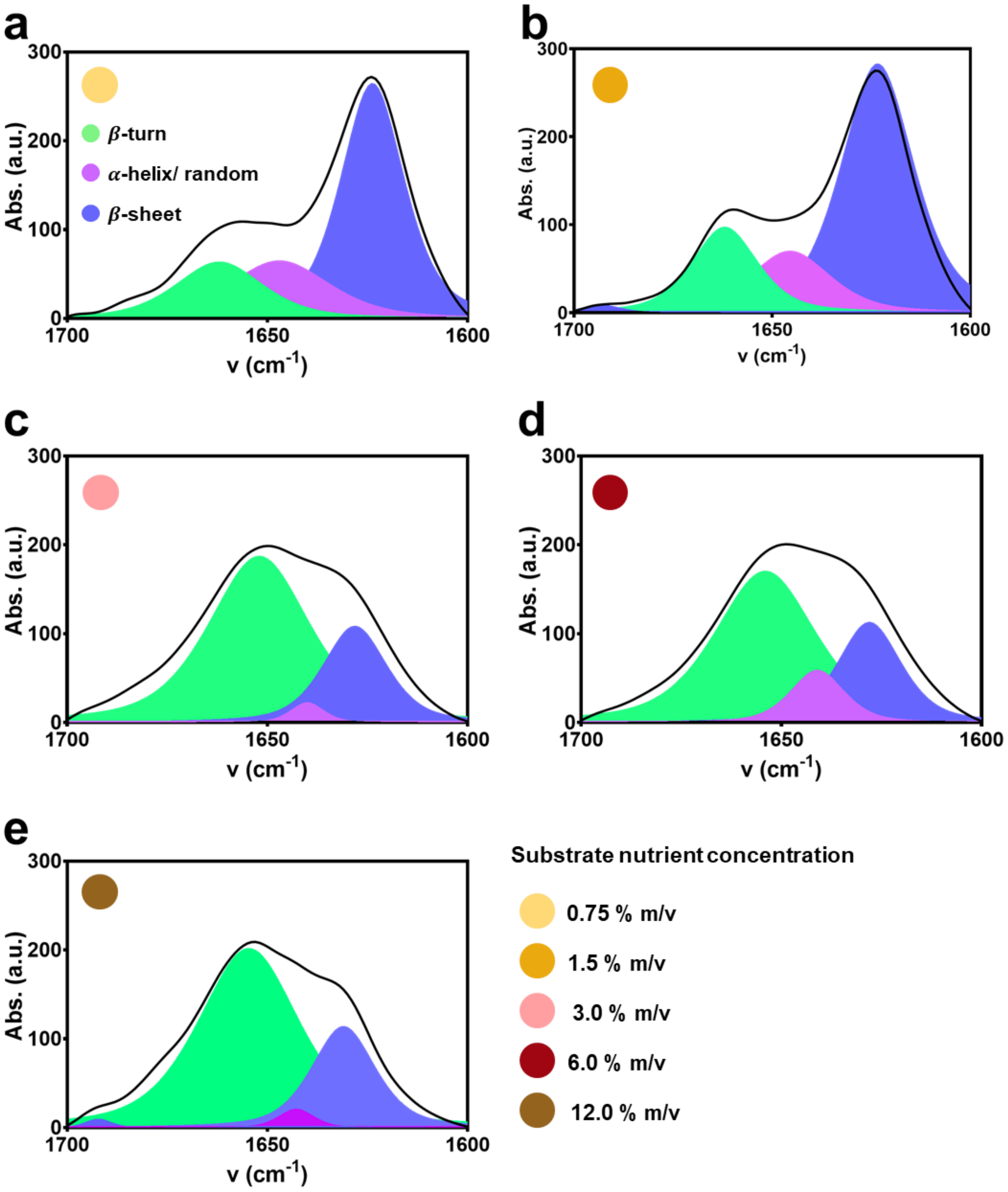

Supplementary Figure 14 Amide I' spectra of curli fibers purified from *E. coli* biofilms. Area-normalized FTIR absorbance spectra in the Amide I' region of the different samples. The spectra were curve-fitted using the number of spectral components identified by second derivative on the Fourier self-deconvoluted spectra. N = 3.

Supplementary Table 2 Secondary structure analysis of the purified curli fibers assessed by ATR-FTIR. The values correspond to Figure 3d. N = 3.

Substrate nutrient concentration (% w/v)	Secondary structure content (%)		
	β - sheet	α - helix/ random	Turns
0.75	58.03 ± 0.41	22.44 ± 0.12	19.82 ± 0.10
1.50	57.33 ± 1.04	29.63 ± 3.84	16.08 ± 1.63
3.00	30.08 ± 5.98	4.87 ± 3.00	65.01 ± 5.74
6.00	25.23 ± 3.66	14.84 ± 10.41	59.82 ± 6.92
12.00	26.72 ± 1.88	3.41 ± 0.78	69.86 ± 2.61

12. Fiber structure by circular dichroism (CD) spectrometry

The purified curli fibers showed differences in their β-sheet structure as described in their CD spectra (**Supplementary Figure 14**). All fibers tested in this experiment were normalized by CsgA monomer concentration, meaning there is the same protein concentration in each sample tested. Because of this, lower signal intensity means lower β-sheet content. Hence, fibers from biofilms grown on agar substrates containing lower nutrient concentration (0.75 and 1.5 % w/v), present high β-sheet content than those from biofilms grown on agar substrates containing higher nutrient concentration (3.0 % w/v or higher). These results are complementary to those of acquired with the ATR-FTIR experiments.

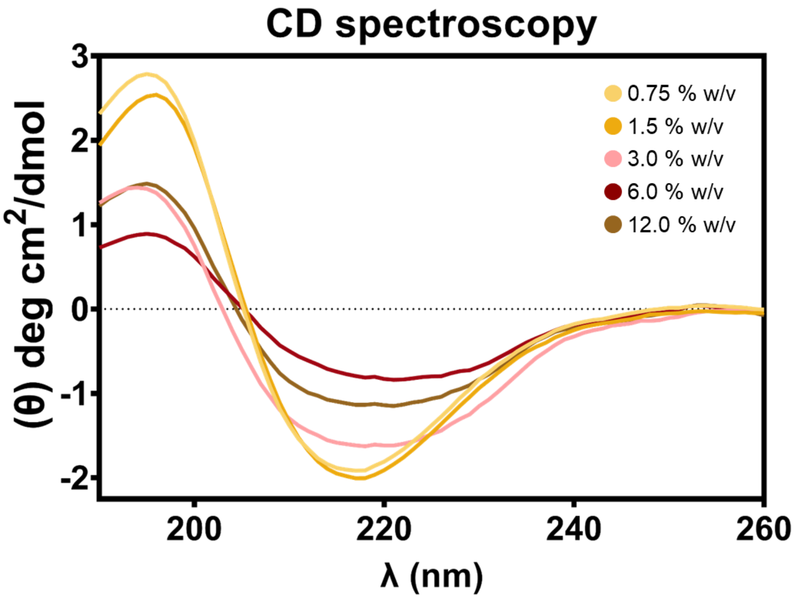

Supplementary Figure 15 CD spectrometry of the purified curli fibers. All fibers were normalized by CsgA monomer concentration. Data come from N = 3 independent biofilm cultures for each condition tested.

13. Fiber polarity

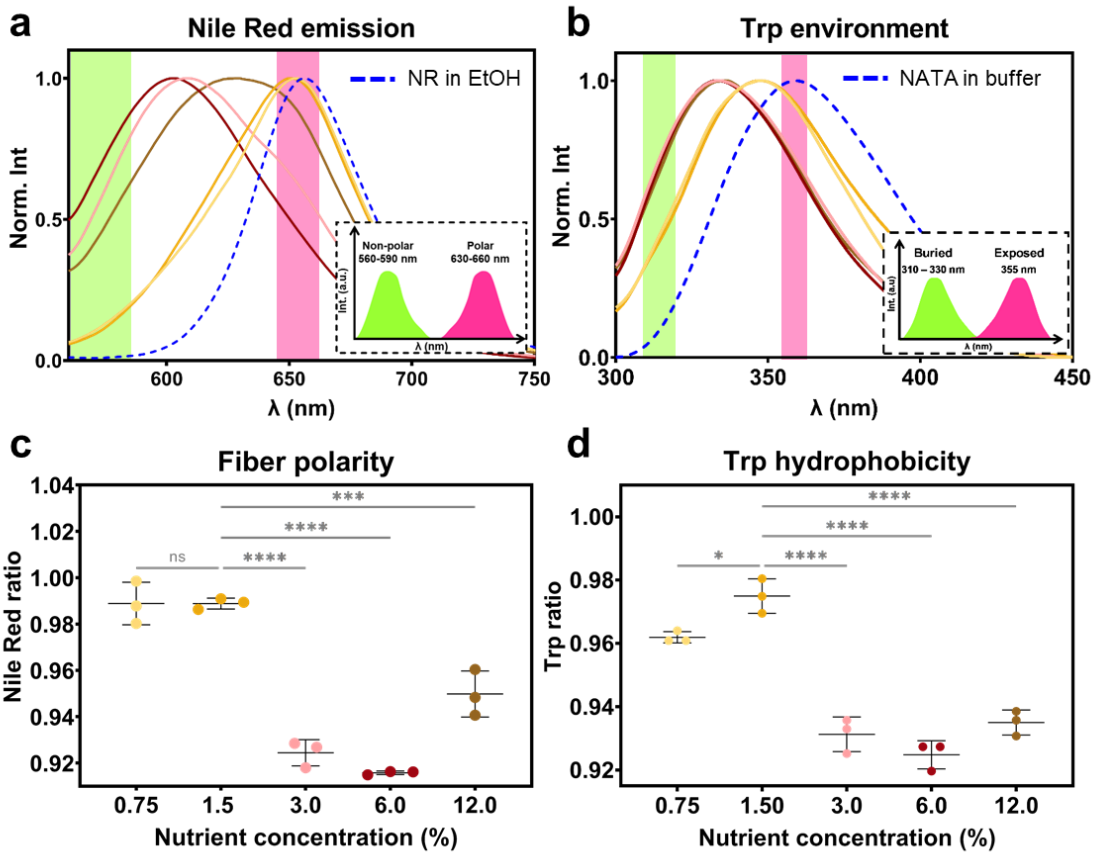

Supplementary Figure 16 Fiber polarity. (a) Fluorescence spectra of fibers stained with Nile Red (NR). The spectra of NR in buffer is represented as references for the emission of NR in a hydrophobic and hydrophilic environment, respectively. A sketch of the position of peaks for the NR in non-polar and polar environments is represented in the inset.[8] The shadowed areas in the main plot indicate these exposure extremes. N=3-4 independent biofilm cultures for each condition tested. (b) Intrinsic fluorescence of the fibers through Trp emission. The spectrum of soluble Trp (NATA) in buffer is represented as reference for the maximum exposure possible of the Trp to the surface (λ_{exc} = 280 nm). A sketch of the position of peaks for buried and exposed Trp is represented in the inset.[9] The shadowed areas in the main plot indicate these exposure extremes. N=3-4 independent biofilm cultures for each condition tested**.** (c) Ratio between the position emission maximum of each condition, taking NR in buffer as a reference. (d) Ratio between the position emission maximum of each condition, taking NATA in buffer as a reference. Data come from N = 3 independent biofilm cultures for each condition tested. The statistical analysis was done with One-way ANOVA (p<0.0001, **** | p<0.001, *** | p<0.01, ** | p<0.05, * | ns = non-significant), where the 1.5 % w/v nutrient concentration condition was used as reference for the post-test multicomparisons.

14. Curli fiber and CsgA subunit

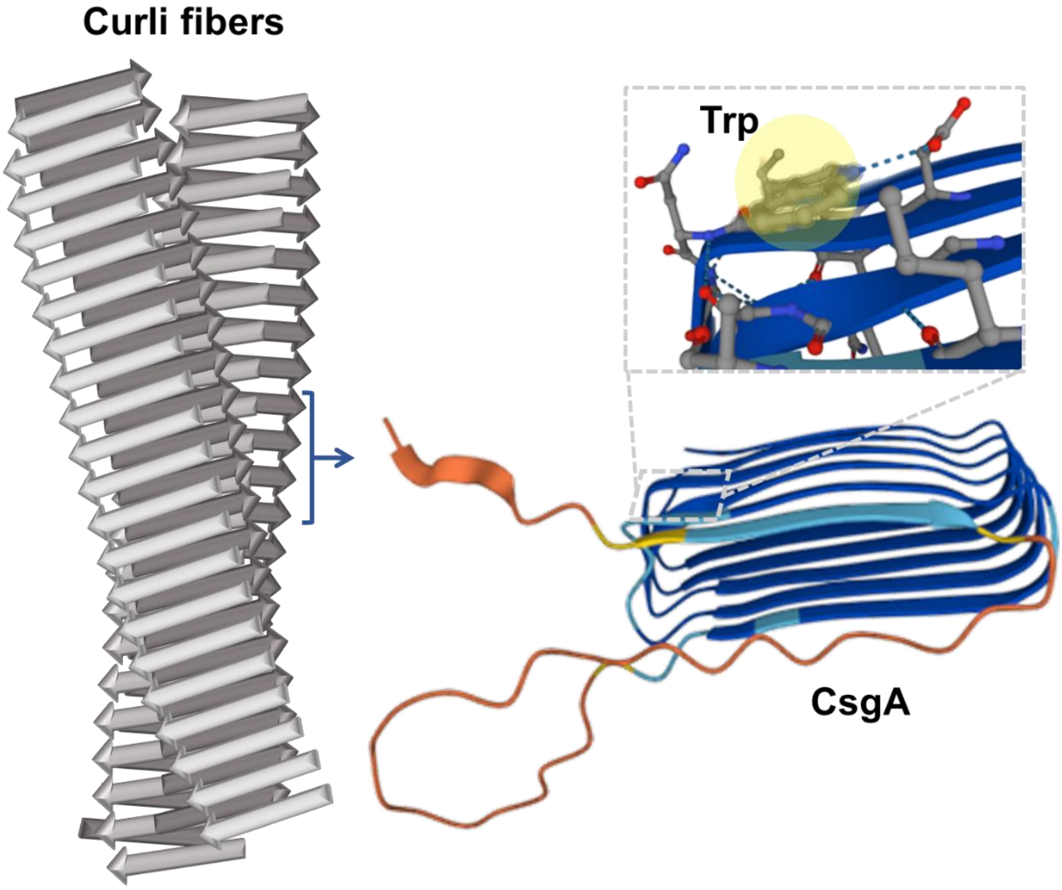

Supplementary Figure 17 Scheme of the composition of curli fibers and the position of the Trp in the CsgA protein.

15. Structure/function relationship between purified curli fibers and biofilm mechanical properties

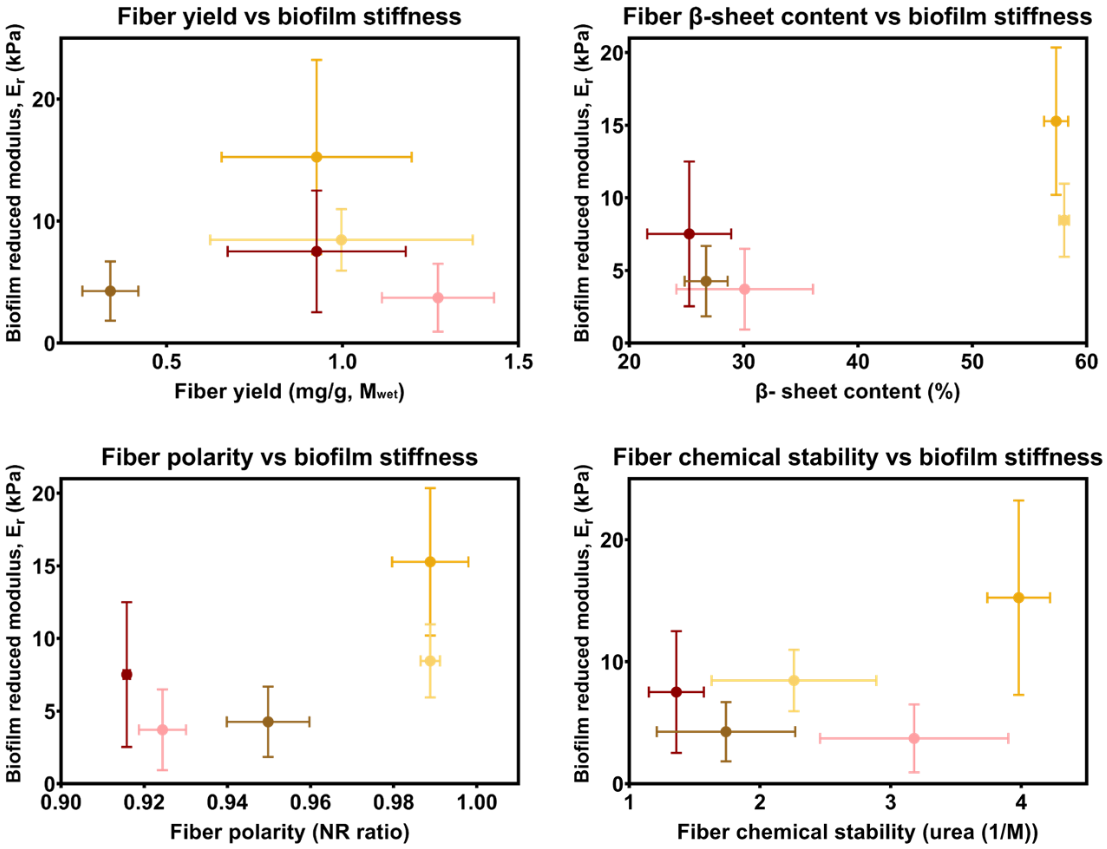

Supplementary Figure 18 Biofilm mechanical properties as a function of different properties of the purified curli fibers.

16. Proof of concept: from purified curli fibers to CsgA to fibers again

We purified the curli fibers from biofilms grown in two different conditions of the biofilm: LB agar substrates containing 1.5% w/v and 6.0% w/v nutrients. We then reduced these fibers to their monomer state (CsgA), and fibrillated them again in various buffers (**Supplementary Figure 19**). To favor clarity, the curli fibers purified from biofilms grown on LB agar substrates containing 1.5% w/v nutrients are “Fiber A” and the curli fibers purified from biofilms grown on LB agar substrates containing 6.0% w/v nutrients are “Fiber B”.

CsgA monomers or polymers were identified by a band of the SDS-PAGE around 15 kDa and two bands around 30 kDa[5], as well as by CD spectroscopy with a peak close to 200 nm[5] (**Supplementary Figure 19 b** and **c**). The increase of ThioT emission indicates that the previously denatured fibers can repolymerize in each of the media tested (**Supplementary Figure 19d**). Fibers grown in LB 1.5% w/v show a slower polymerization process for the monomers of fiber A and fiber B compared to the other conditions tested. Fibers grown in buffer or LB 6.0% w/v have similar polymerization curves.

At the end of the polymerization experiment, each sample was centrifuged to separate the resulting fibers from the unpolymerized CsgA (supernatant) and restained with ThioT for fiber identification (**Supplementary Figure 19e**). Because of the low yield in each sample, the concentration of fibers measured by ThioT emission was not normalized to any concentration. In all cases, the ThioT emission spectra observed displayed the shape expected in the presence of amyloid fibers in the sample. Moreover, electron microscopy (TEM) confirmed the existence of fibers (**Supplementary Figure 19f**).

The ATR-FTIR spectra of the newly polymerized fibers in each medium showed spectra with peaks in similar positions to those of their respective original fibers (before denaturation with FA) (**Supplementary Figure 19g**). Upon detailed analysis of these spectra, band assignment and secondary structure fitting suggest the structure of the newly polymerized fibers in each media follow the same trend as the structure of their respective original fibers (Fiber A and Fiber B, respectively), amidst the statistical differences observed (**Supplementary Figure 19h**). These exploratory results suggest that depending on the environmental cues bacteria are under, they might express the CsgA monomer with a different secondary structure, thus defining different structural conformations of the mature curli fibers.

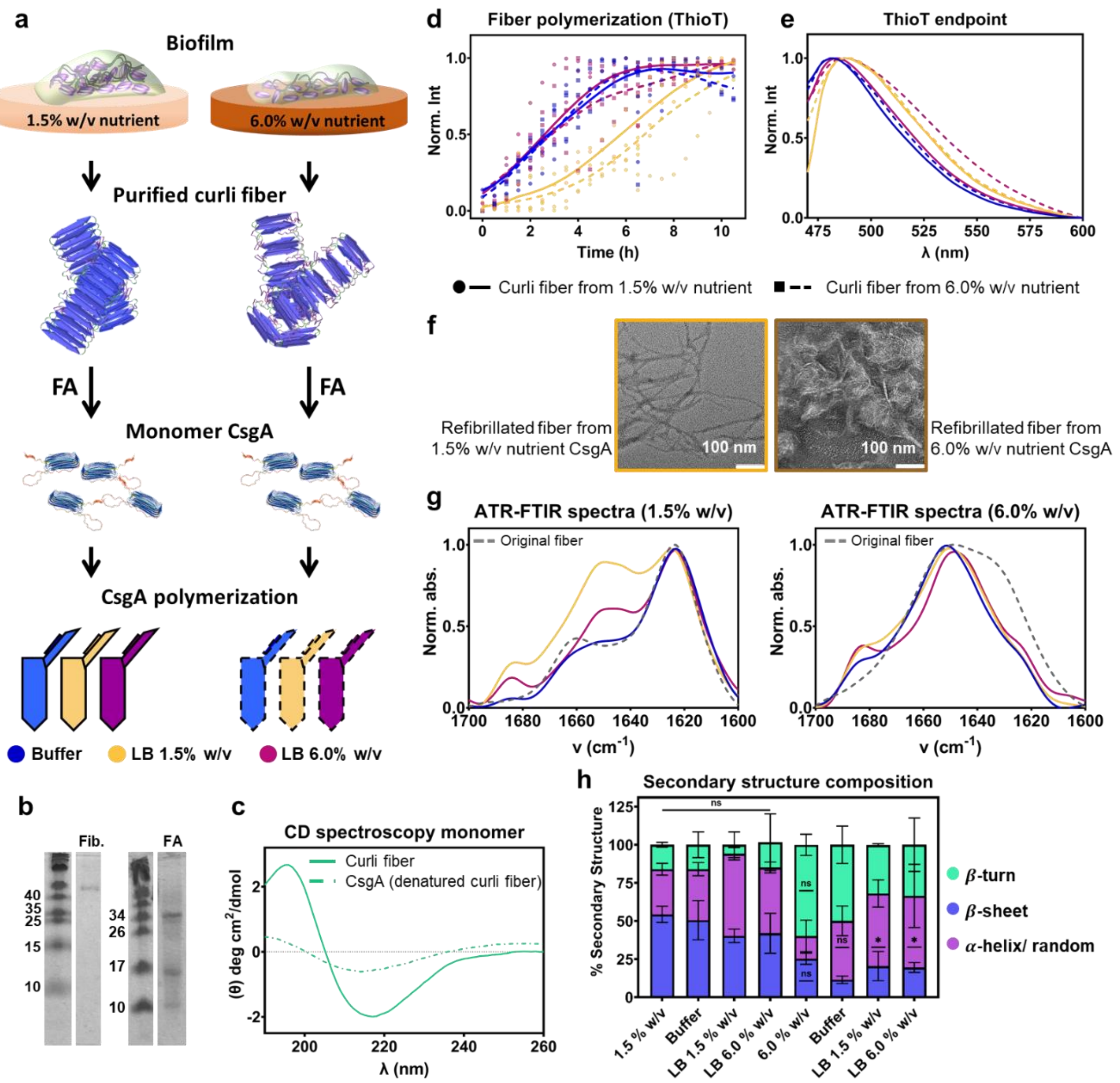

Supplementary Figure 19 Curli fiber denaturation and characterization of refibrillated fibers. (a) Schematic workflow. (b) Coomassie stain SDS-PAGE purified curli fibers (Fib.) and of samples after fiber denaturation with formic acid (FA). Molecular size markers (in kDa) are indicated in the corresponding ladder. (c) CD spectra of the purified curli fibers and of the CsgA monomer after denaturation. (d) CsgA polymerization followed by ThioT emission. (e) ThioT fluorescence emission of the dye bound to the fibers after CsgA polymerization in (d). (f) TEM images of the polymerized fibers. Scale bar = 100 nm. (g) Amide I' spectra of polymerized fibers. Area-normalized FTIR absorbance spectra in the Amide I' region of the different samples. The spectra were curve-fitted using the number of spectral components identified by second derivative on the Fourier self-deconvoluted spectra. (h) Distribution of the three types of secondary structure in the polymerized fibers. The data was obtained from the Amide I' region of each spectra. The statistical analysis was done with One-way ANOVA ($p<0.0001$, **** | $p<0.001$, *** | $p<0.01$, ** | $p<0.05$, * | ns = non-significant), where the 1.5 % w/v nutrient concentration condition was used as reference for the post-test multicomparisons. All data came from two independent denaturation experiments, except fiber polymerization that was done four times for each condition (duplicate of each denaturation experiment).

References

1. Bertasa, M. *et al.* A study of non-bounded/bounded water and water mobility in different agar gels. *Microchem. J.* **139**, 306–314 (2018).

2. Schindelin, J. *et al.* Fiji: An open-source platform for biological-image analysis. *Nat. Methods* **9**, 676–682 (2012).

3. Buchanan, R. E. Life Phases in a Bacterial Culture. *J. Infect. Dis.* **23**, 109–125 (1918).

4. Presnov, E. V. Synchronization of cell division. *J. Biol. Syst.* **7**, 213–223 (1999).

5. Chapman, M. R. *et al.* Role of Escherichia coli curli operons in directing amyloid fiber formation. *Science (80-.).* **295**, 851–855 (2002).

6. Yakupova, E. I., Bobyleva, L. G., Vikhlyantsev, I. M. & Bobylev, A. G. Congo Red and amyloids: History and relationship. *Biosci. Rep.* **39**, (2019).

7. Reichhardt, C. & Cegelski, L. The Congo red derivative FSB binds to curli amyloid fibers and specifically stains curliated E. Coli. *PLoS One* **13**, 1–9 (2018).

8. Jameson, D. M. Introduction to Fluorescence. *Principles of Fluorescence Spectroscopy* 1–26 at https://doi.org/10.1007/978-0-387-46312-4_1 (2006).

9. Lakowicz, J. R. *Principles of fluorescence spectroscopy, 3rd Principles of fluorescence spectroscopy, Springer, New York, USA, 3rd edn, 2006. Principles of fluorescence spectroscopy, Springer, New York, USA, 3rd edn, 2006.* (2006). doi:10.1007/978-0-387-46312-4.
